# Supplementary material for: Structural and mechanistic basis for redox sensing by the cyanobacterial transcription regulator RexT
Source: Commun Biol. 2022 Mar 28;5:275. doi: 10.1038/s42003-022-03226-x (PMC8960804; doi:10.1038/s42003-022-03226-x)
Supplement: Supplementary file 2 — Supplementary Information [file 42003_2022_3226_MOESM2_ESM.pdf]

Structural and Mechanistic Basis for Redox Sensing by the Cyanobacterial Transcription Regulator RexT

Bin Li[1], Minshik Jo[1], Jianxin Liu[1], Jiayi Tian[1], Robert Canfield[1-2], and Jennifer Bridwell-Rabb[1]

Corresponding Author: jebridwe@umich.edu

[1]Department of Chemistry, University of Michigan, Ann Arbor, MI 48109
[2]Current Address: Department of Microbiology and Immunology, University of Colorado Anschutz Medical Campus, Aurora, CO 80045

Supplementary Figures and Tables:

Supplementary Fig. 1. RexT is a dimer.
Supplementary Fig. 2. Structure comparison of RexT with ArsR-SmtB superfamily members.
Supplementary Fig. 3. The ability of RexT to bind DNA is not impacted by the presence of metal ions.
Supplementary Fig. 4. RexT resembles the ArsR-SmtB protein NolR.
Supplementary Fig. 5. An electrophoretic mobility shift assay for RexT and its variants allows for probing interaction with DNA.
Supplementary Fig. 6. Two chloride ions serve as a mimic of the DNA backbone in the crystal structure of RexT.
Supplementary Fig. 7. Mass spectrometry reveals key insights into disulfide bond formation.
Supplementary Fig. 8. Circular dichroism experiments reveal a similar architecture in the presence and absence of H_2O_2.
Supplementary Fig. 9. Crystal packing likely prevents disulfide bond formation in chain A.
Supplementary Fig. 10. A significant conformational change occurs in the RexT structure following oxidation.
Supplementary Fig. 11. The structure of RexT reveals key structural features for responding to H_2O_2.
Supplementary Fig. 12. The HTH ArsR-SmtB-type DNA-binding domain and related protein families were used to generate a sequence similarity network (SSN).
Supplementary Fig. 13. Sequence alignment of previously characterized members of ArsR-SmtB family.
Supplementary Fig. 14. The sub-sequence similarity network with genomic neighborhood information annotated for the RexT cluster.
Supplementary Fig. 15. Sequence alignment of RexT homologs reveals complete conservation of Cys41.
Supplementary Fig. 16. A rooted phylogentic tree for RexT homologs from three orders of Cyanobacteria.
Supplementary Fig. 17: Alignment of RexT with CyeR shows conservation of Cys41.
Supplementary Fig. 18. Comparison of RexT with other ArsR-SmtB members in different states suggests possible mechanism of regulation.

Supplementary Table 1. The sequence of DNA duplex probe used for EMSA.
Supplementary Table 2. Binding constants of RexT and its variants to DNA duplex fluorescence probe.
Supplementary Table 3. Primers used for mutagenesis.
Supplementary Table 4. Key ArsR-SmtB members highlighted in the sequence similarity network.
Supplementary Table 5. 105 sequences of RexT-like regulators that control Trx expression.

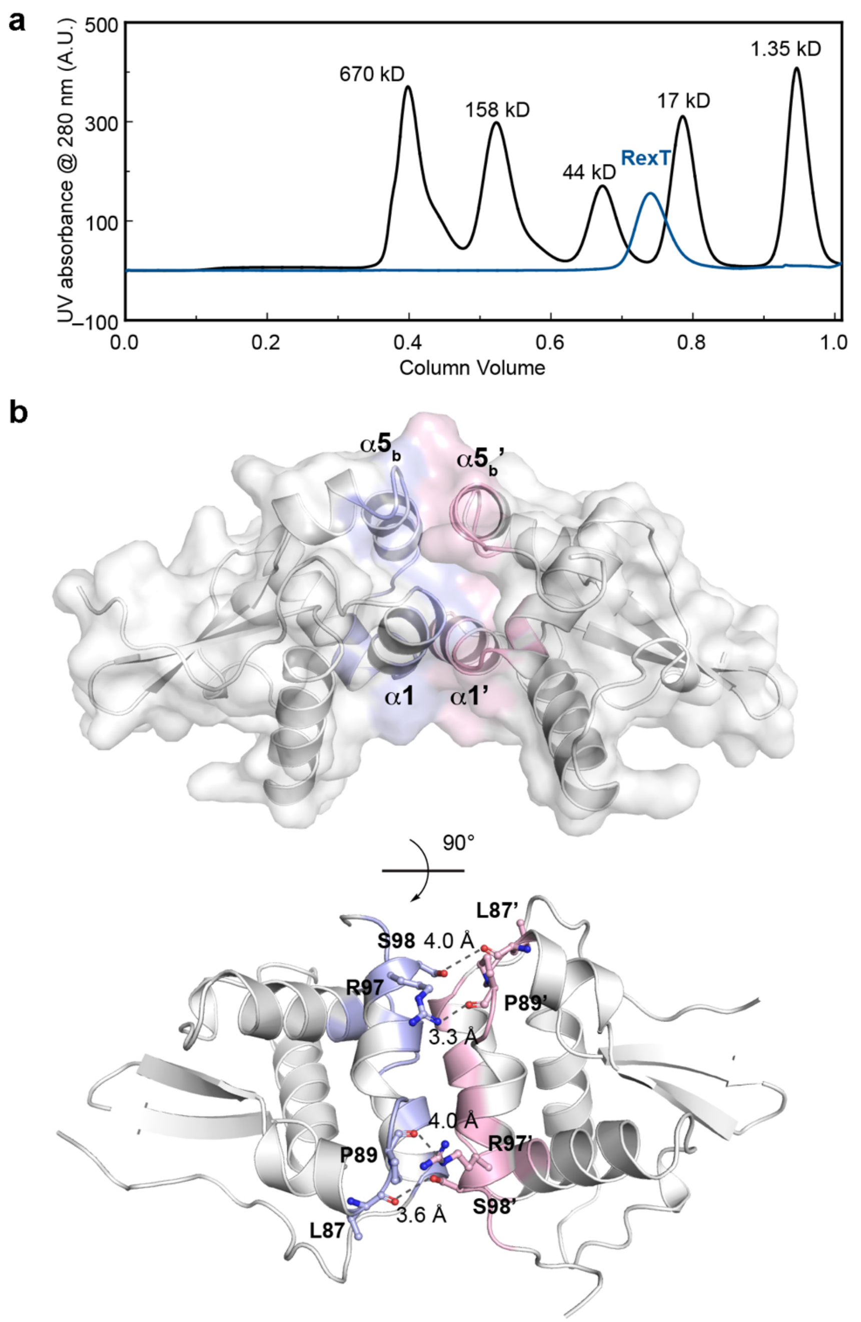

Supplementary Fig. 1. RexT is a dimer. (a) Size exclusion chromatography shows that RexT exists in a dimeric form in solution. The protein standard (Bio-Rad) contains thyroglobin (670 kD), γ-globin (158 kD), ovalbumin (44 kD), myoglobin (17 kD) and vitamin B12 (1.35 kD). Tag-free RexT based on our purification protocol after TEV-cleavage has a calculated monomeric molecular weight of 12.8 kD based on the amino acid sequence. RexT elutes between ovalbumin and myoglobin, suggesting that it exists in a dimeric form (25.6 kD as a dimer). (b) The dimer interface of RexT is made up by the coiled-coil interaction between the α1 helices, and between the $\alpha 5_b$ helices. The interacting residues are identified by the ePISA server[1] and are colored in light blue on chain A and light pink on chain B. The interacting residues are Leu14, Pro15, Leu18, Tyr19, Leu21, Gly22, Asp23, Pro24, Arg26 and Leu27 on the α1 helix and Leu87, Phe88, Pro89, Gly90, Leu91, Ala94, Val95, Arg97, Ser98, Ala99, Gln100 on the $\alpha 5_b$ helix. Polar interactions at the dimeric interface are indicated with dashed lines and the distances are labeled.

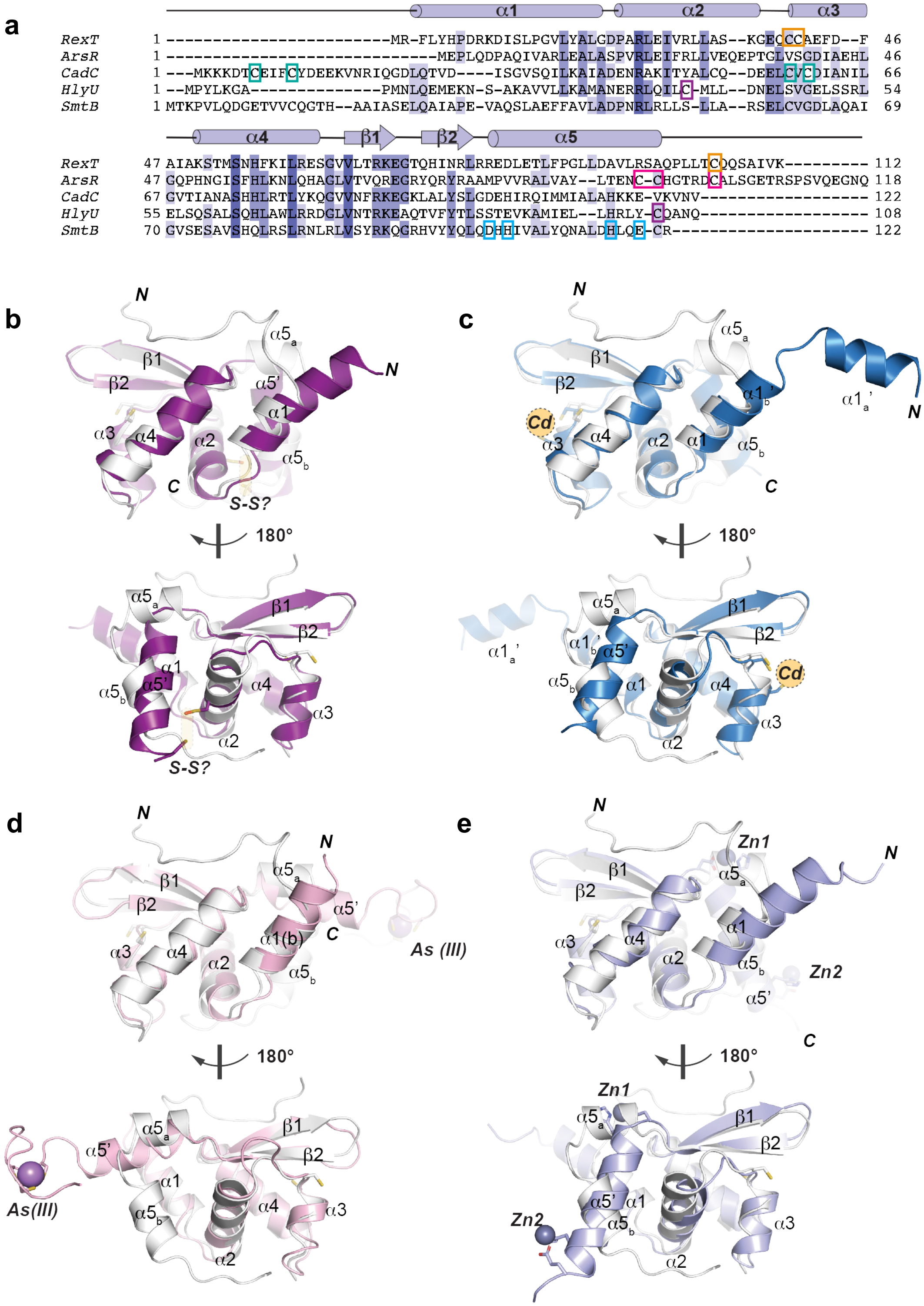

Supplementary Fig. 2. Structure comparison of RexT with ArsR-SmtB superfamily members. (a) Sequence alignment of four ArsR-SmtB transcription factors identified by the Dali server[2] with RexT shows a well conserved wHTH architecture. Secondary structure labeling is based on the consensus. Colored boxes highlight functional residues that could bind an environmental stimulator, such as a metal ion (green and blue), an arsenite ion (pink), form a disulfide bond in RexT (orange), or an unknown species (purple). (b) RexT (gray) is overlaid with *Vc*HlyU[3] (PDB ID: 4K2E, purple). In *Vc*HlyU Cys38 on the α2 helix is in the sulfenic acid form and is pointing towards Cys104, suggesting that disulfide bond formation may be important to regulation. The root mean squared deviation (rmsd) is 1.82 Å over 396 atoms. (c) RexT (gray) is overlaid with *Sa*CadC[4] (PDB ID: 1U2W, blue). *Sa*CadC uses a regulatory cadmium site (dashed circle).

The rmsd is 1.45 Å over 404 atoms. (d) RexT (gray) is overlaid with *Af*ArsR[5] (PDB ID: 6J05, pink). *Af*ArsR uses three Cys residues on the C-terminus to bind As^{3+} (purple sphere). The rmsd is 1.33 Å over 365 atoms. (e) RexT (gray) is overlaid with *Se*SmtB[6] (PDB ID: 1R22, light purple). In this protein, two Zn^{2+} (gray sphere) ions at the dimer interface are found coordinated to Asp and His residues from the α5 helix of one subunit, and His and Glu residues from the other subunit (only a monomer is shown here). The rmsd is 2.05 Å over 426 atoms. In all panels, the overlay of RexT was performed in COOT[7] using the SSM superimpose feature to align the models based on Cα positions and visualized by PyMOL. *Af*, *Acidithiobacillus ferrooxidans*; *Se*, *Synechococcus elongatus* PCC 7942; Sa, *Staphylococcus aureus*; Vc, *Vibrio cholerae.*

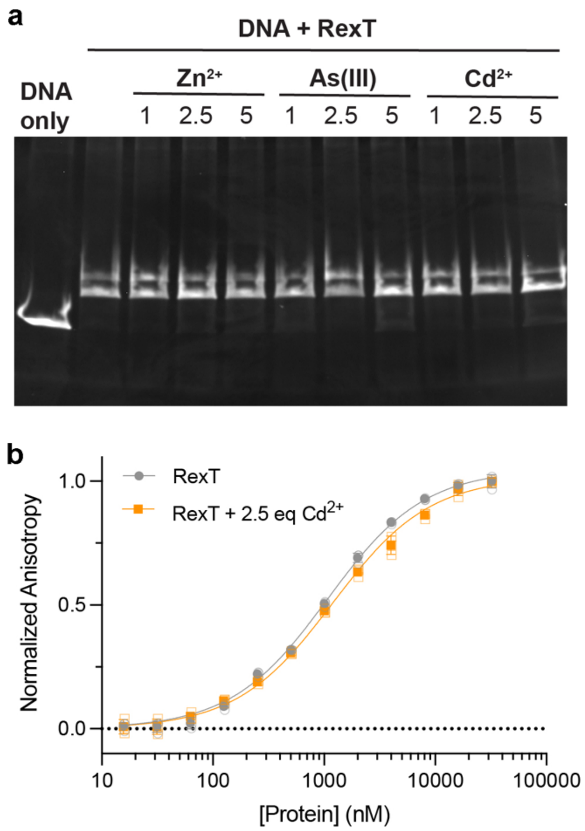

Supplementary Fig. 3. The ability of RexT to bind DNA is not impacted by the presence of metal ions*.* (a) A DNA probe (20 nM) was incubated with RexT (100 nM) and 1, 2.5, or 5 equivalents of As(III), Cd^{2+}, and Zn^{2+}. This electrophoretic mobility shift assay (EMSA) showed that RexT binds to DNA similarly in the absence (lane 2) and presence of the different tested metal ions (lanes 3-11). (b) A labeled DNA probe shows changes in fluorescence anisotropy following the addition of RexT. These differences allowed for calculation of the K_d for RexT in the presence (orange) and absence (gray) of Cd^{2+} (*see* Supplementary Table 2). As shown in panel A, adding a metal ion to RexT in a 2.5-fold excess does not markedly change its ability to bind DNA. Each data point for the experiments is shown as an open shape. In panel b, data was measured using n=3 independent experiments and is presented with the individual measurements (open shapes) and as the mean value of these measurements ± SD (closed shapes). Source data are provided as a Source Data file.

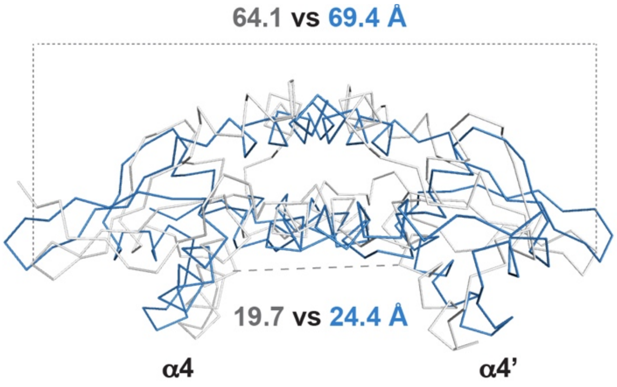

Supplementary Fig. 4. RexT resembles the ArsR-SmtB protein NolR. An overlay of RexT with NolR[8] (PDB: 4ON0) based on their Cα atoms. RexT is shown in light gray and NolR is shown in blue. Dashed and dotted lines show the distance between mid-points of the recognition helix and between the tip of the "wing" (dark gray label for RexT and blue label for NolR).

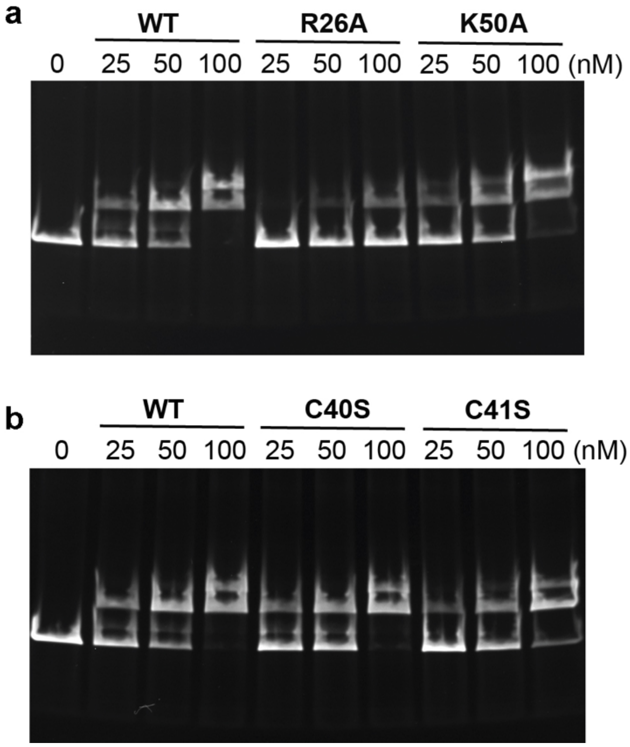

Supplementary Fig. 5. An electrophoretic mobility shift assay for RexT and its variants allows for probing interaction with DNA. (a) The DNA probe (20 nM) was incubated with 25, 50 and 100 nM of wild type (WT) dimeric RexT and the R26A, K50A variants at room temperature for 30 min. Both variants impact the ability of RexT to interact with DNA. (b) The DNA probe (20 nM) was also incubated with 25, 50 and 100 nM WT dimeric RexT and the C40S and C41S variants at room temperature for 30 min. The C40S variant doesn't show much difference in DNA-binding relative to WT RexT whereas C41S showed decreased DNA-binding affinity. In both panels, the 0 nM lane is the negative control where no protein was added.

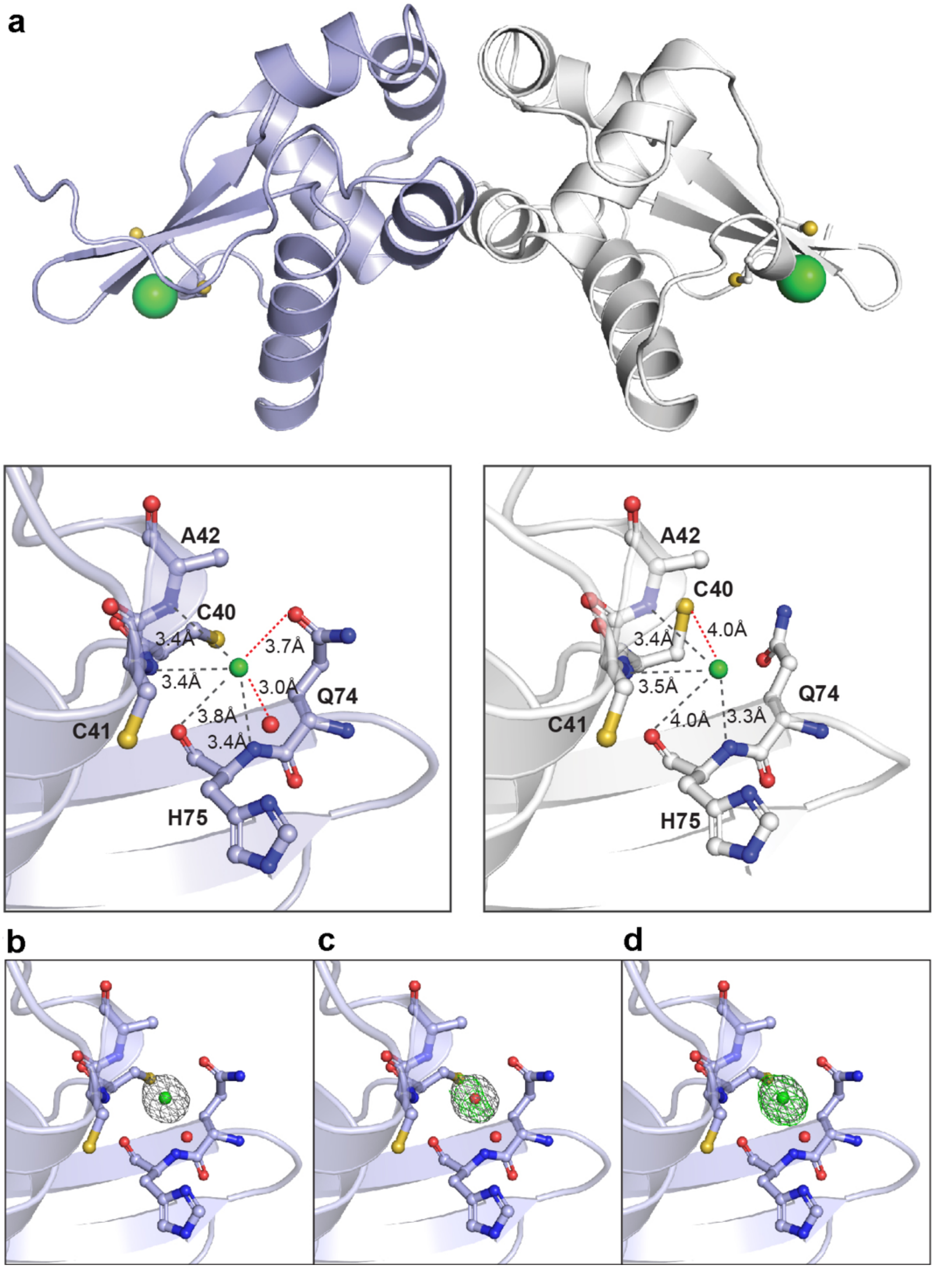

Supplementary Fig. 6. Two modeled chloride ions serve as a mimic of the DNA backbone in the crystal structure of RexT. (a) Two chloride ions are found in the crystal structure of reduced RexT, between Cys40 and Cys41. The chloride ions interact with the protein backbones, side chains, and water molecules in chain A (blue, bottom left panel) and chain B (white, bottom right panel). Dashed gray lines show conserved interactions in both chains A and B, including the interaction with the Cys41 backbone amide nitrogen, Ala42 backbone amide nitrogen and His75 backbone carbonyl oxygen and amide nitrogen. Red dotted lines show subunit-specific interactions. In chain A, the chloride interacts with a nearby water and the side chain oxygen of Asn74. In chain B, the chloride may have a weak interaction with the sulfur atom of Cys40. (b) The 2Fo-Fc (gray) and Fo-Fc (green/red) electron density map calculated for the refined structure of RexT around the modeled chloride ion. These maps are shown contoured at 1.0σ and ±3.0σ, respectively. (c) The 2Fo-Fc (gray) and Fo-Fc (green/red) electron density map calculated after chloride was omitted from the structure of RexT and water was modeled in its place. These maps are shown contoured at 1.0σ and ±3.0σ, respectively. (d) The 2Fo-Fc (gray) and Fo-Fc (green/red) electron density map calculated after the chloride ion was omitted from the structure of RexT. These maps are shown contoured at 1.0σ and ±3.0σ, respectively.

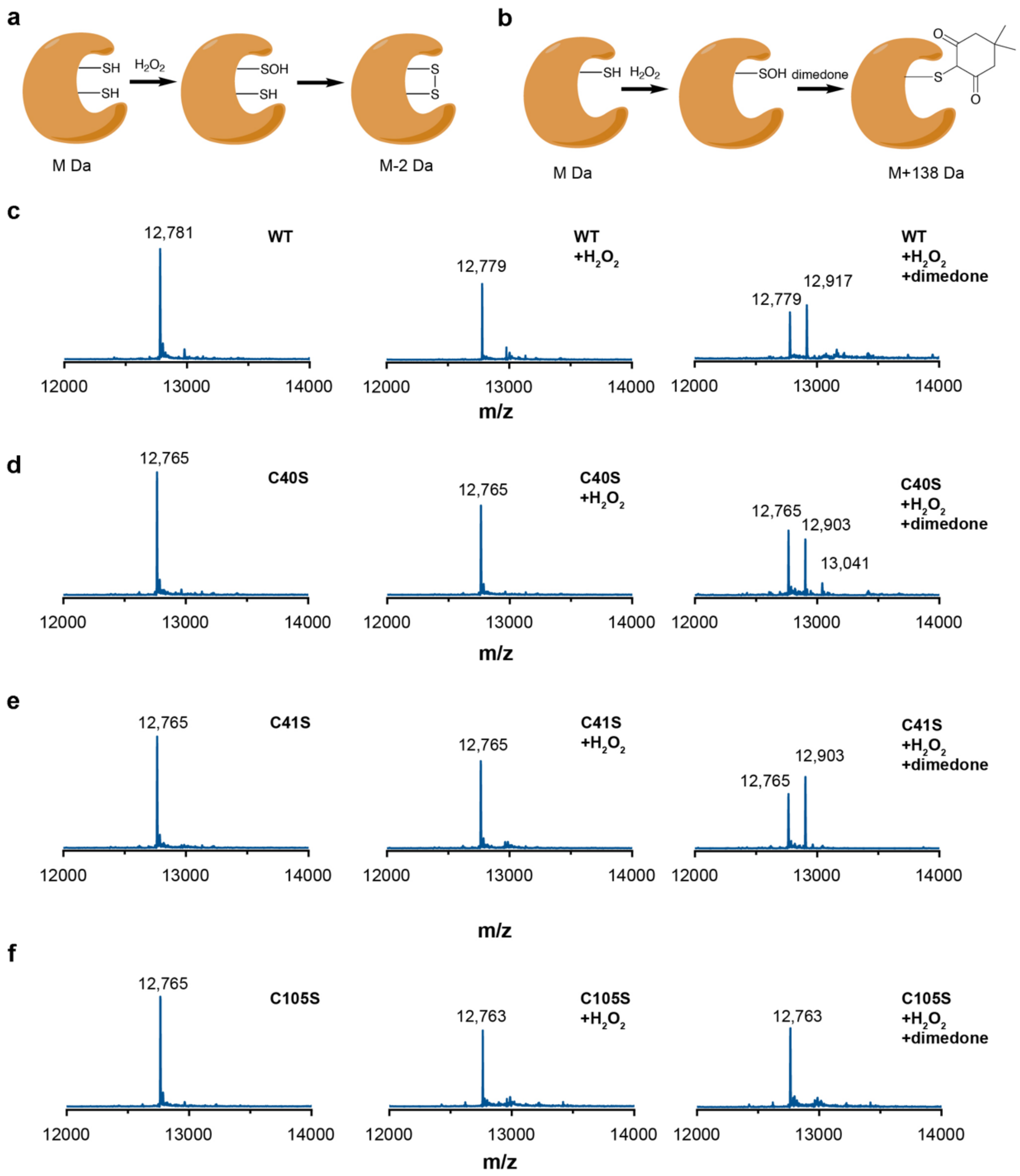

Supplementary Fig. 7. Mass spectrometry reveals key insights into disulfide bond formation. (a) To probe the identity of the disulfide bond forming Cys residues, mass spectrometry experiments were performed on wild-type (WT) RexT and each of its Cys variants in the presence of H_2O_2. The mass of RexT with a disulfide bond should be 2 Da less than when a disulfide bond is not formed. (b) As disulfide bond formation proceeds through formation of a sulfenic acid moiety, mass spectrometry was also used to look for incorporation of the small molecule dimedone into RexT following the addition of H_2O_2. This experiment was performed to identify the peroxidatic Cys residue. Incorporation of one molecule of dimedone into RexT results in a 138 Da increase in the mass of the protein. (c) The mass spectrometry experiments are shown for WT RexT in the absence of H_2O_2 (left), presence of H_2O_2 (middle), and presence of H_2O_2 and dimedone (right). These experiments show formation of a disulfide bond (middle), formation of a disulfide bond and the incorporation of one molecule of dimedone (right). (d) Mass spectrometry of the C40S RexT variant shows no formation of a disulfide bond (middle) and the incorporation of two molecules of dimedone (right). (e) Mass spectrometry of the C41S RexT variant shows no formation of a disulfide bond (middle) and the incorporation of one molecule of dimedone (right). (f) Mass spectrometry of the C105S RexT variant shows formation of a disulfide bond (middle), formation of a disulfide bond and no incorporation of dimedone (right).

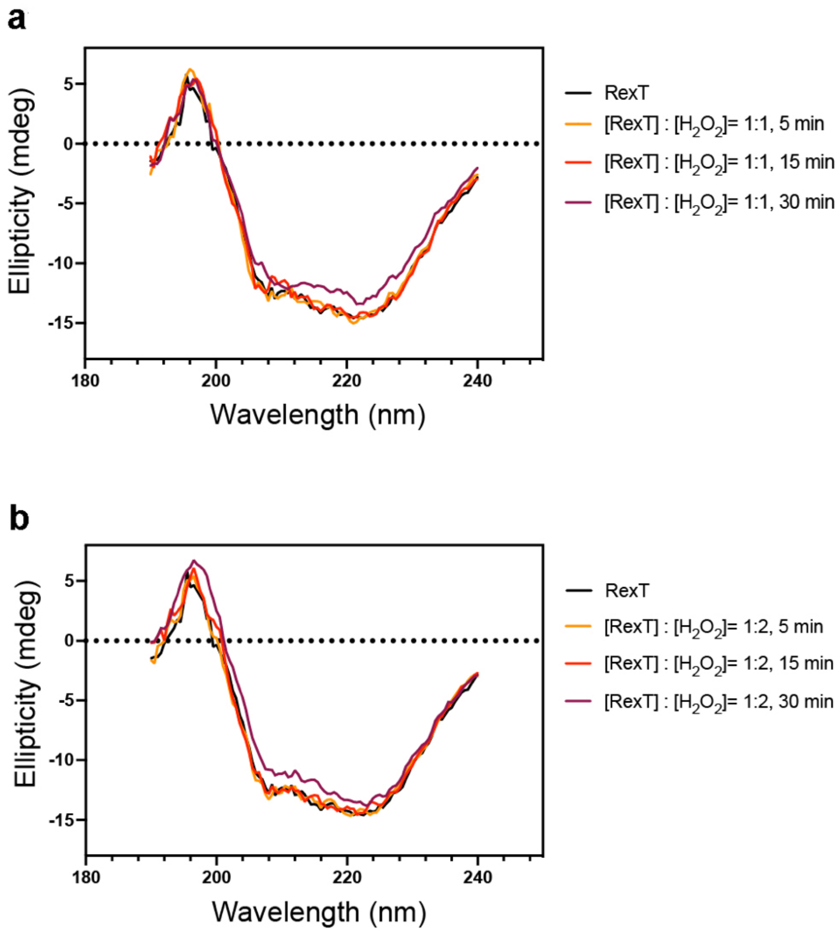

Supplementary Fig. 8. Circular dichroism experiments reveal that RexT has a similar architecture in the presence and absence of H_2O_2. (a) Circular dichroism (CD) data shows that the RexT (black) when combined with one equivalent of H_2O_2 does not remarkably change in structure over time (orange, red, and purple). (b) Similarly, CD data shows that the RexT (black) when combined with two equivalents of H_2O_2 does not remarkably change in structure over time (orange, red, and purple).

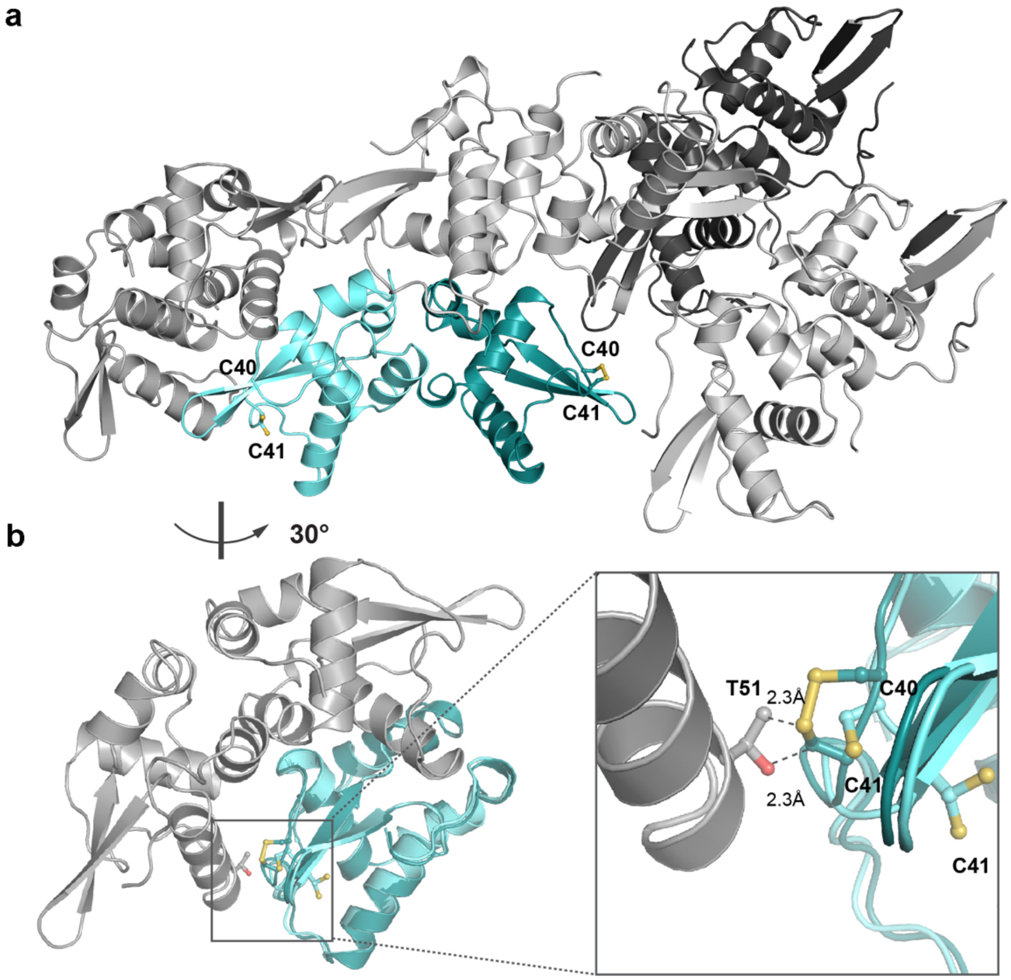

Supplementary Fig. 9. Crystal packing likely prevents disulfide bond formation in chain A. (a) The oxidized structure of RexT (chain A is cyan and chain B is dark cyan) with its crystal symmetry mates (gray). In chain B, there is space for a disulfide bond to be formed between Cys40 and Cys41. In chain A, a symmetry molecule is packed up against where the bond would be formed. (b) Chain B of RexT is overlaid with chain A to show the clash that would occur upon disulfide bond formation.

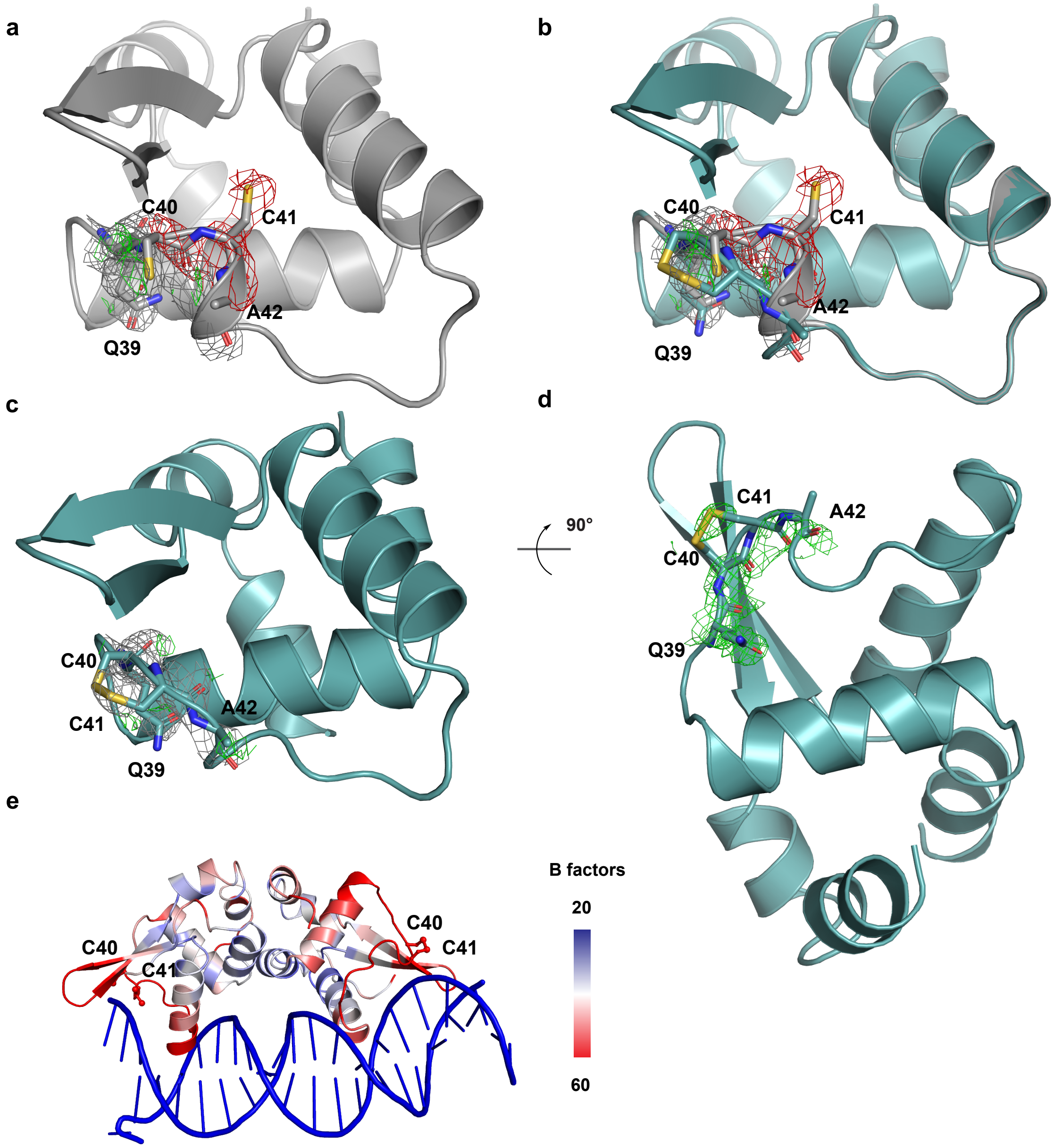

Supplementary Fig. 10. A significant conformational change occurs in the RexT structure following oxidation. (a) The reduced structure of RexT (gray) has a different conformation than the oxidized in the α3 helical region. Shown in this panel are the 2Fo-Fc (gray) and Fo-Fc (green/red) electron density maps contoured at 1.0σ and ±2.0σ, respectively, when the structure of the reduced RexT is used as a model for the oxidized RexT data. (b) Modeling the oxidized structure (cyan) with a disulfide bond is a better fit of the data. Again, the 2Fo-Fc (gray) and Fo-Fc (green/red) electron density maps contoured at 1.0σ and ±2.0σ, respectively. (c) The refined 2Fo-Fc (gray) and Fo-Fc (green/red) electron density maps contoured at 1.0σ and ±2.0σ, respectively, around the oxidized structure in the region of the modeled disulfide bond are shown. (d) A Fo-Fc omit (green) electron density map contoured at ±2.0σ is shown around the α3 helical region after it was omitted from the refined structure of oxidized RexT. (e) Alignment of the oxidized RexT structure with the DNA-bound NolR structure (PDB: 4ON0)[8] shows that the wHTH regions of RexT that interact with DNA are highly flexible.

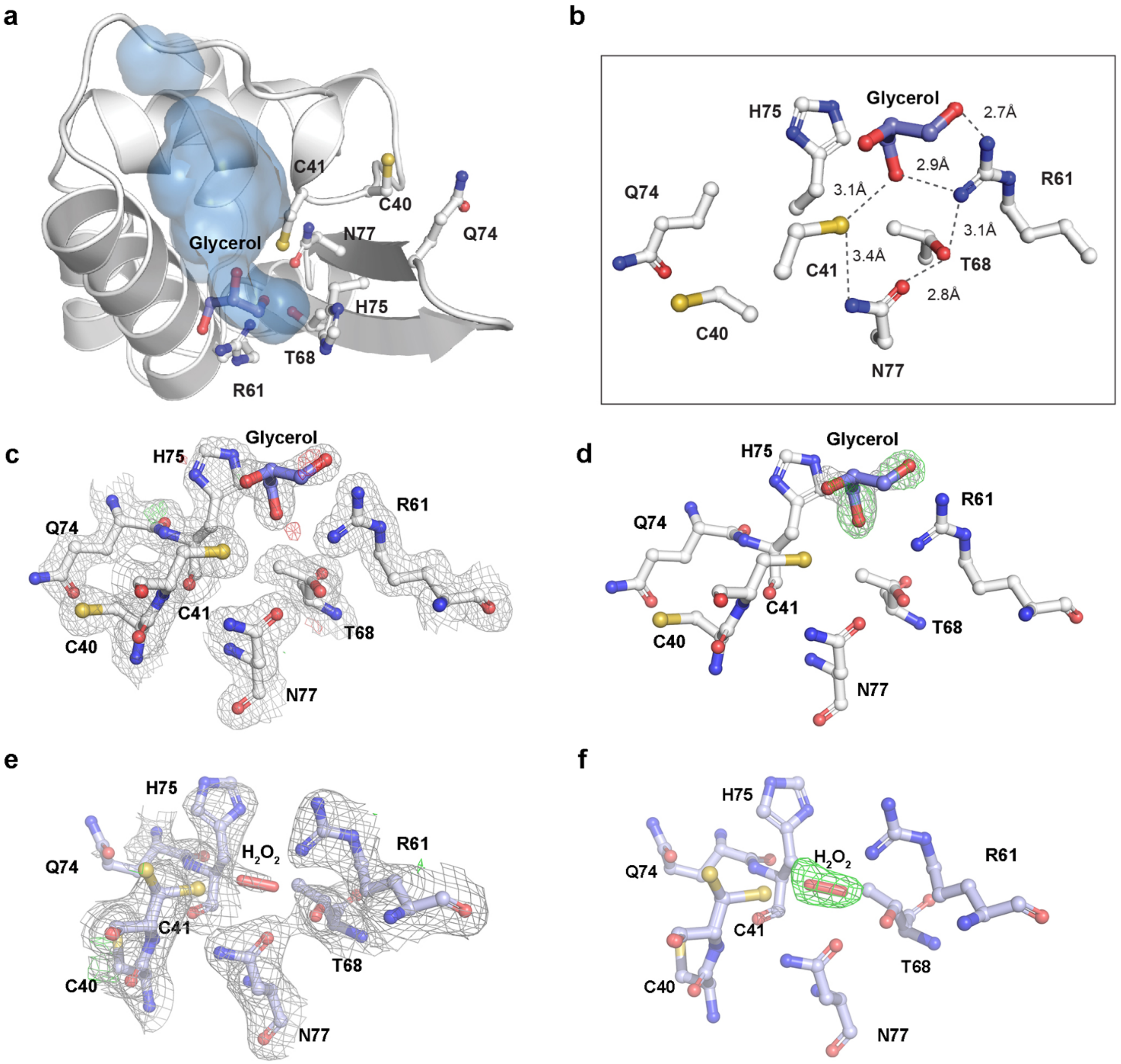

Supplementary Fig. 11. The structure of RexT reveals key structural features for responding to H_2O_2. (a) A surface cavity (blue) is identified that leads from the surface of RexT to the Cys 41 residue. The calculated cavity of chain B in the reduced RexT structure contains a glycerol molecule from the cryoprotectant. (b) The bottom of the calculated cavity that surrounds Cys41 contains residues arranged in a way that suggests they are important players in activation of H_2O_2. (c) The electron density maps for the residues and glycerol molecule shown in panel B are displayed. The refined 2Fo-Fc (gray) and Fo-Fc (green/red) electron density maps contoured at 0.9σ and ±3.0σ, respectively. (d) The 2Fo-Fc and Fo-Fc electron density map calculated after glycerol was omitted from the structure of reduced RexT. These maps are shown contoured at 0.9σ and ±3.0σ, respectively. (e) The 2Fo-Fc (gray) and Fo-Fc (green/red) electron density maps for the residues and H_2O_2 molecule from Fig. 5B are displayed contoured at 0.9σ and ±3.0σ, respectively. (f) The 2Fo-Fc and Fo-Fc electron density map calculated after H_2O_2 was omitted from chain A of the oxidized RexT structure are displayed contoured at 0.9σ and ±3.0σ, respectively.

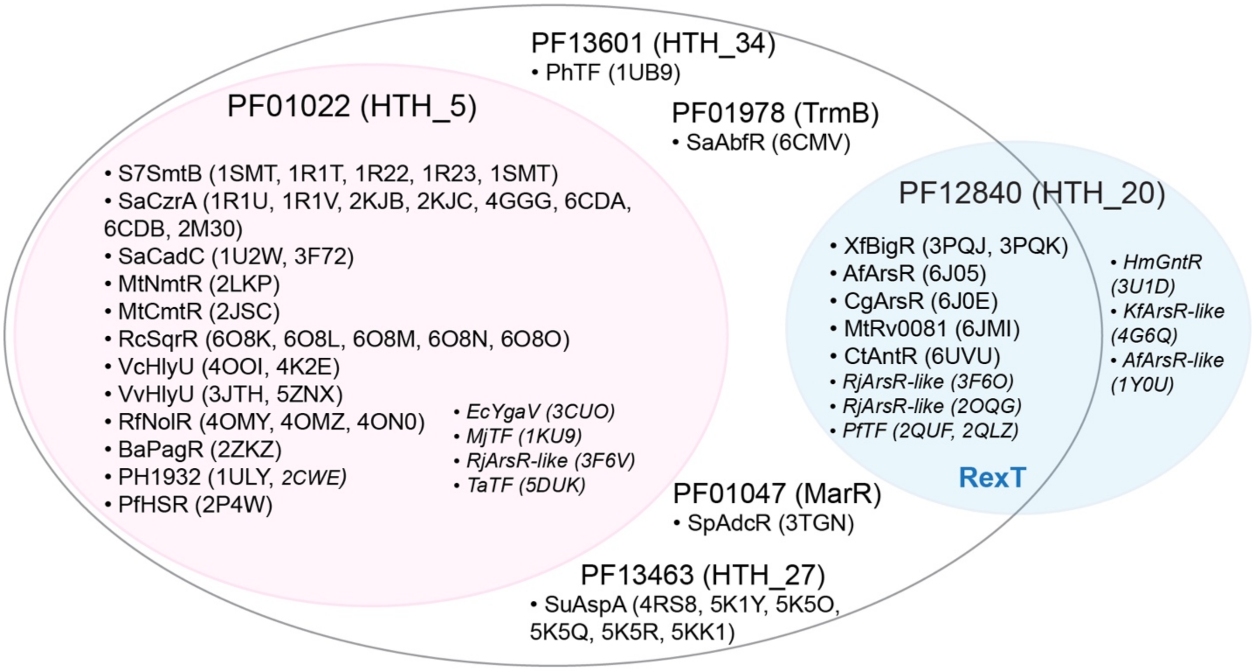

Supplementary Fig. 12. The HTH ArsR-SmtB-type DNA-binding domain and related protein families were used to generate a sequence similarity network (SSN). Proteins and their structures that have an annotated “HTH ArsR-type DNA-binding domain” (IPR001845, large oval shape). This classification encompasses a few protein families, predominantly PF01022 (HTH_5, shaded in pink) and PF12840 (HTH_20, shaded in blue). Notably a few proteins that belong to PF12840 do not have the annotated IPR001845 domain (shaded in blue, outside of the large oval). Proteins and PDB IDs shown in italics do not have an accompanying publication.

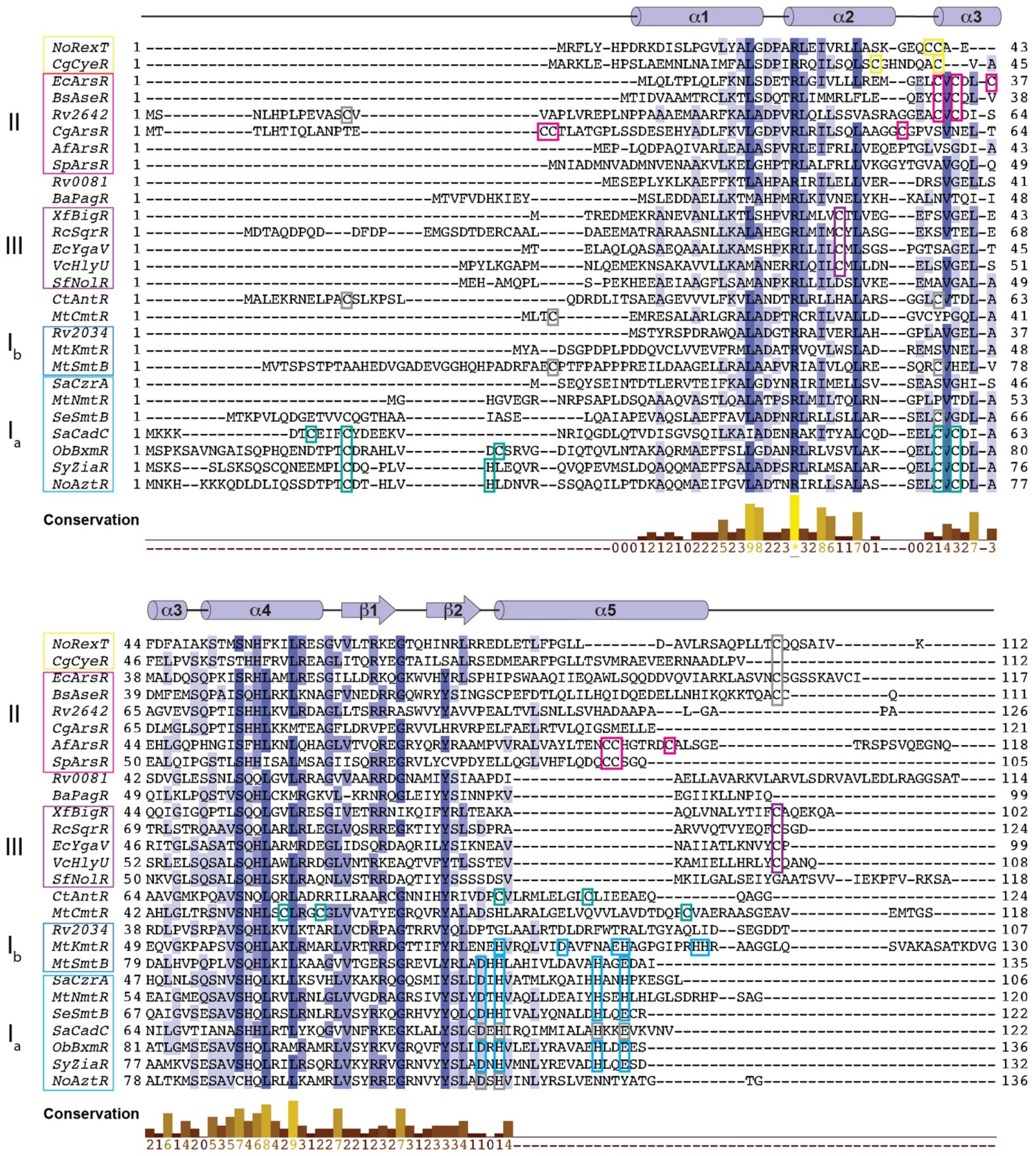

Supplementary Fig. 13. Sequence alignment of previously characterized members of the ArsR-SmtB family. Conserved secondary structures are labeled as cylinders (α-helices) and arrows (β-strands) based on the consensus. Key amino acid residues that are implicated in regulatory process are colored: blue, His/Asp/Glu-rich motif for binding of "hard" metal ions such as Ni^{2+} and Zn^{2+}; green, Cys-rich motif for binding "soft" metal ions such as Pb^{2+} and Cd^{2+}; magenta, Cys-rich motif for binding of arsenite or methyl-arsenite; purple, key Cys residues that are involved in redox processes to bind RSS or an unknown species; yellow, key Cys residues that are involved in the ROS response; gray, residues that resembles above-mentioned conserved motifs but do not exhibit any function and are likely the remnant of evolution. The residues responsible for regulation in NolR, *Rv*0081, *Rv*2034 and *Ba*PagR are unknown.

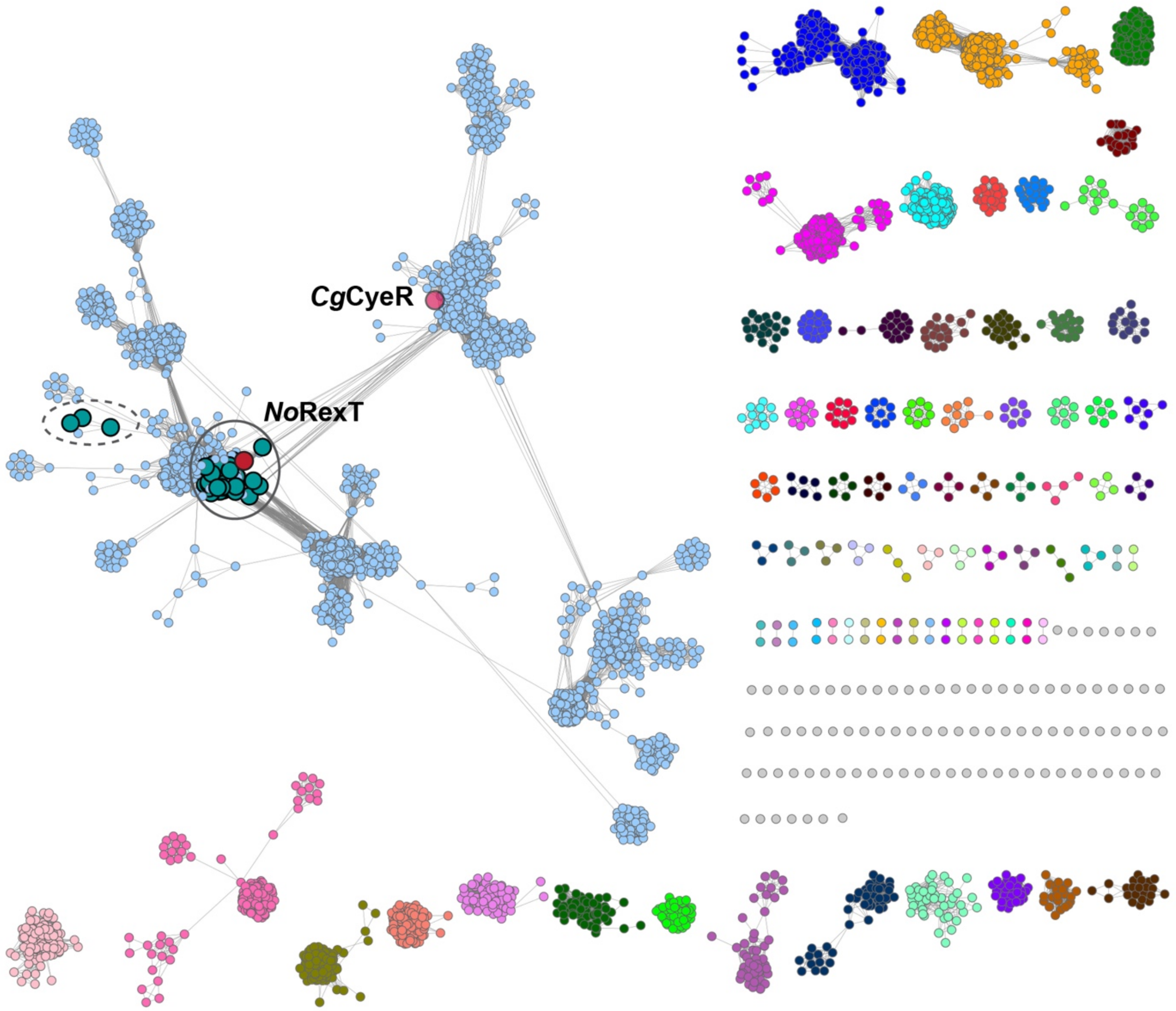

Supplementary Fig. 14. The sub-sequence similarity network with genomic neighborhood information annotated for the RexT cluster. The network is a subset of the network shown in Fig. 6 (the RexT cluster) and consists of 7074 sequences of RexT homologs (the 7085 sequences from Fig. 6 were submitted to the server). It is analyzed in Cytoscape at the cut-off value of e^{-32}. The network is further annotated by the EFI-genome neighborhood tool[9,10] by checking the ten or three genes upstream and downstream of the RexT homologs. The nodes are color-coded based on the numbering of the multi-node clusters in order of decreasing number of sequences. The Pfam for thioredoxin (PF00085) was used as a query to identify its co-occurrence with the RexT homologs. Of interest to this work, in 121 and 108 instances, a thioredoxin gene was found to be within the ten or three-gene neighborhood of RexT homologs, respectively. However, in only 105 of the identified instances is a thioredoxin gene adjacent to a RexT homolog (large teal nodes). 102 of the 105 RexT homologs are from Cyanobacteria (circled with a solid line), two are from unclassified bacteria and one is from Abditibacteria (circled with a dashed line). RexT from *Nostoc* sp. PCC 7120 (*No*RexT) is highlighted in dark red. *Cg*CyeR is found in a different cluster in this network.

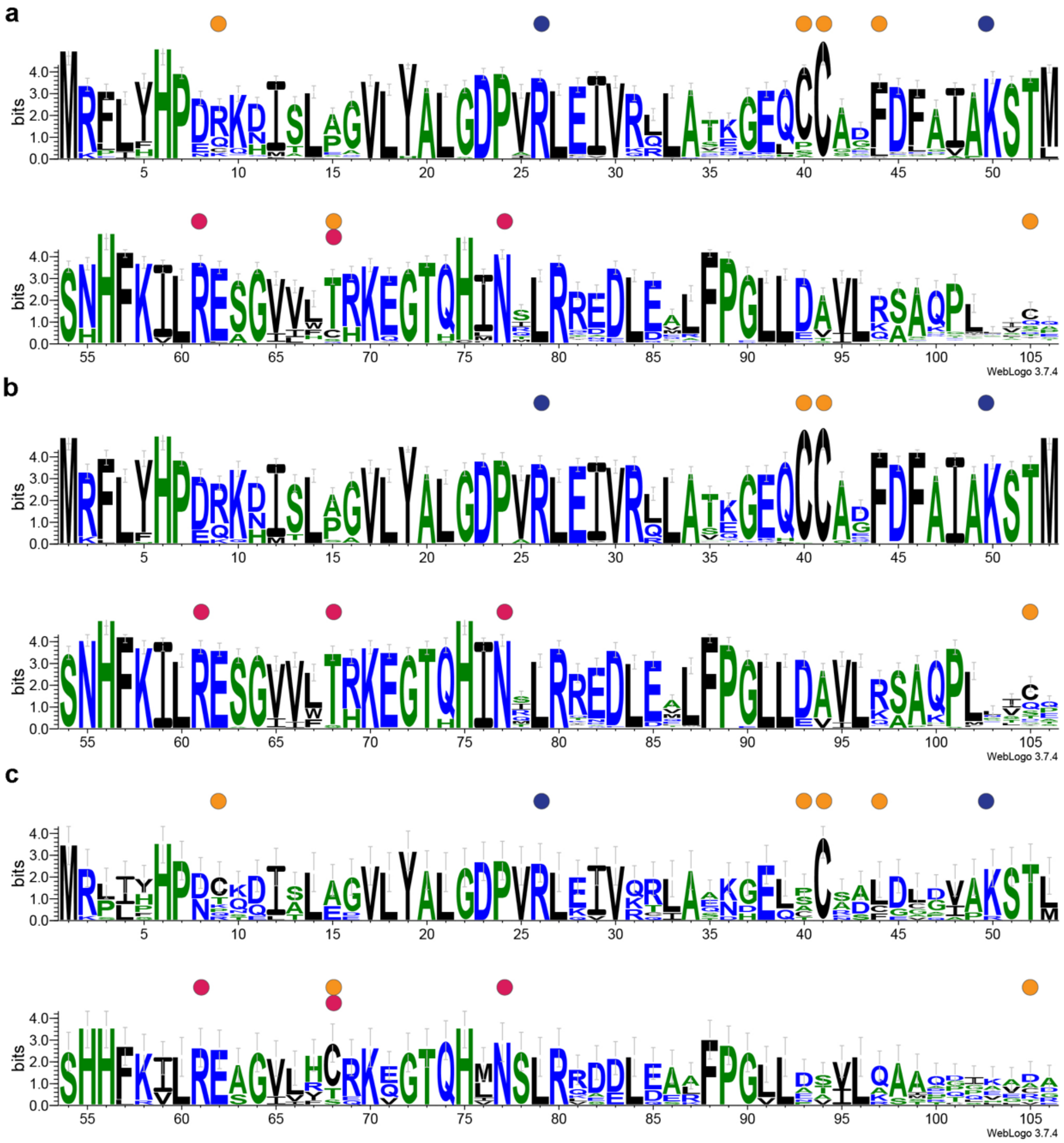

Supplementary Fig. 15. Sequence alignment of RexT homologs reveals complete conservation of Cys41. Sequences were aligned by Clustal W and visualized by WebLogo 3[11] for the first 106 amino acids positions. Orange dots indicate positions where a Cys residue occurs, even at positions of lower occurrence. Dark blue and magenta dots highlight conserved DNA-binding and H_2O_2 activating residues discussed in the main text. (a) The alignment of 104 RexT homologs that were identified adjacent to a thioredoxin gene reveals that the Cys41 residue is completely conserved. In addition, Arg26, and Lys50, which were shown in this work to be involved in DNA binding are completely conserved. The RexT homolog from *Acaryochloris marina* 11017 (UniProt ID: B0C3H5) has an N-terminal truncation of about 25 amino acids and was omitted in the WebLogo visualization. (b) The alignment of 84 RexT homologs from *Nostocales* reveals the conservation of Cys40 and Cys41. Only one homolog from *Tolypothrix* sp. NIES-4075 (UniProt ID: A0A218QHS2) does not contain Cys40. (c) The sequence alignment of 11 homologs from the *Synechococcales* and Oscillatoriales shows the conservation of Cys41, with occurrence of Cys residues also being found at positions 9, 40, 44, 68, 105.

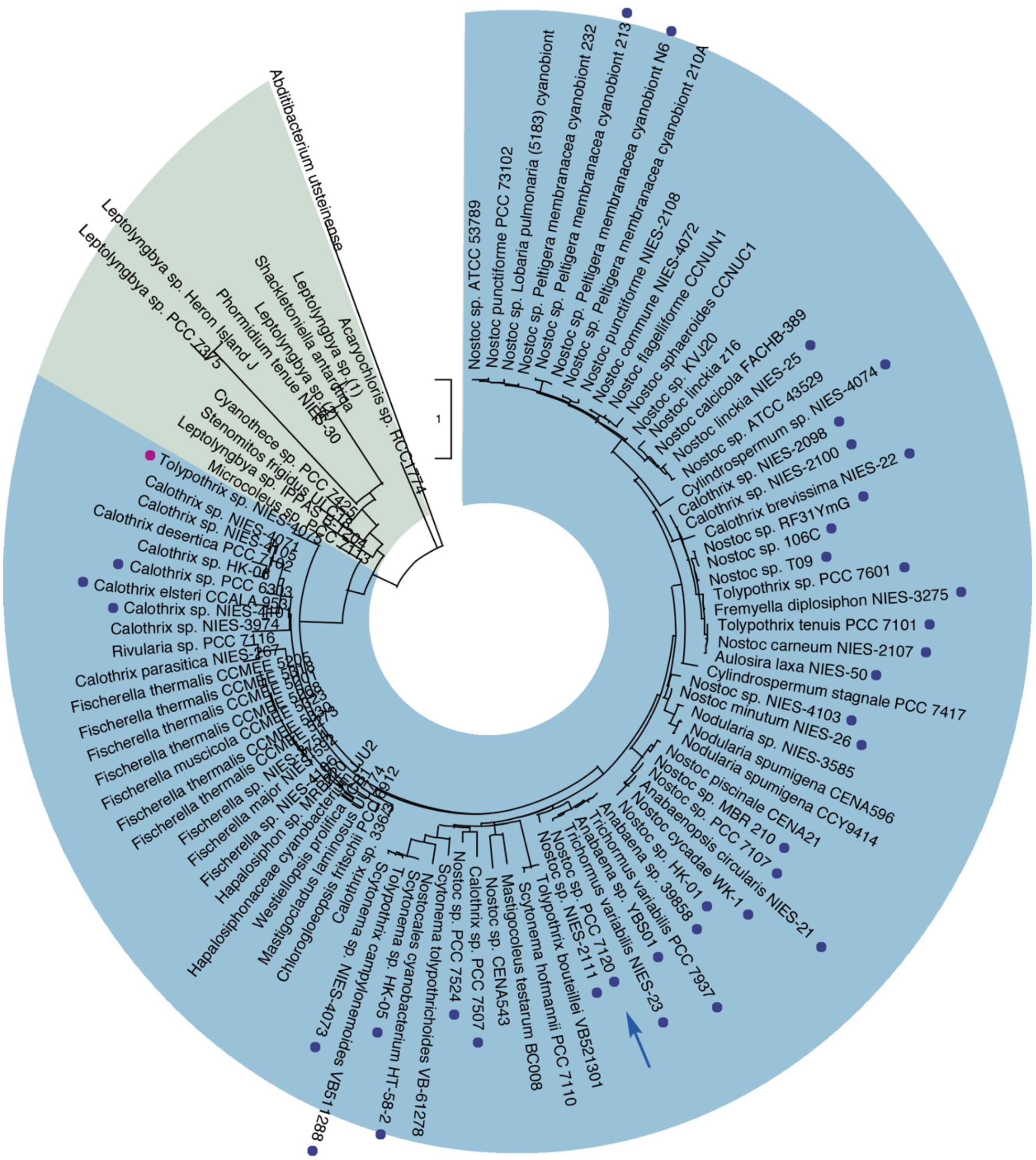

Supplementary Fig. 16. A rooted phylogentic tree for RexT homologs from three orders of Cyanobacteria. This analysis was conducted in MEGA X[12,13] and involved 96 RexT homologs. The RexT sequence from *Abditibacterium utsteinense* (UniProt ID: A0A2S8SU85) was used as the outgroup. The evolutionary history was inferred by using the Maximum Likelihood method and JTT matrix-based model[14]. The tree with the highest log likelihood (-4801.45) is shown. Initial tree(s) for the heuristic search were obtained automatically by applying Neighbor-Join and BioNJ algorithms to a matrix of pairwise distances estimated using the JTT model, and then selecting the topology with superior log likelihood value. A discrete Gamma distribution was used to model evolutionary rate differences among sites (5 categories (+*G*, parameter = 0.3614)). The tree is drawn to scale, with branch lengths measured in the number of substitutions per site. The 11 RexT homologs from *Synechococcales* and *Oscillatoriales* are colored in green and the 84 RexT homologs from *Nostocales* are colored in blue. The RexT homolog from *Tolypothrix* sp. NIES-4075 that does not contain Cys40 is highlighted by a pink dot, and RexT homologs from *Nostocales* that contain an additional Cys105 are highlighted by a blue dot. RexT from *Nostoc* sp. PCC 7120 studied in this work is indicated by an arrow.

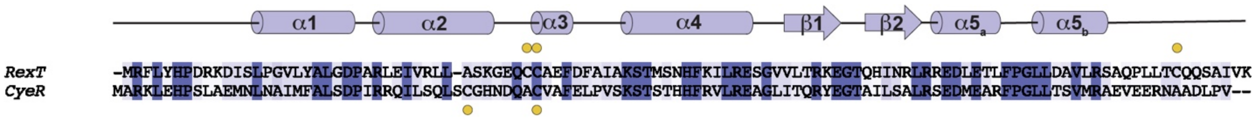

Supplementary Fig. 17. Alignment of RexT with CyeR shows conservation of Cys41. Yellow dots highlight the position of Cys. The secondary structure illustration is based on RexT structure.

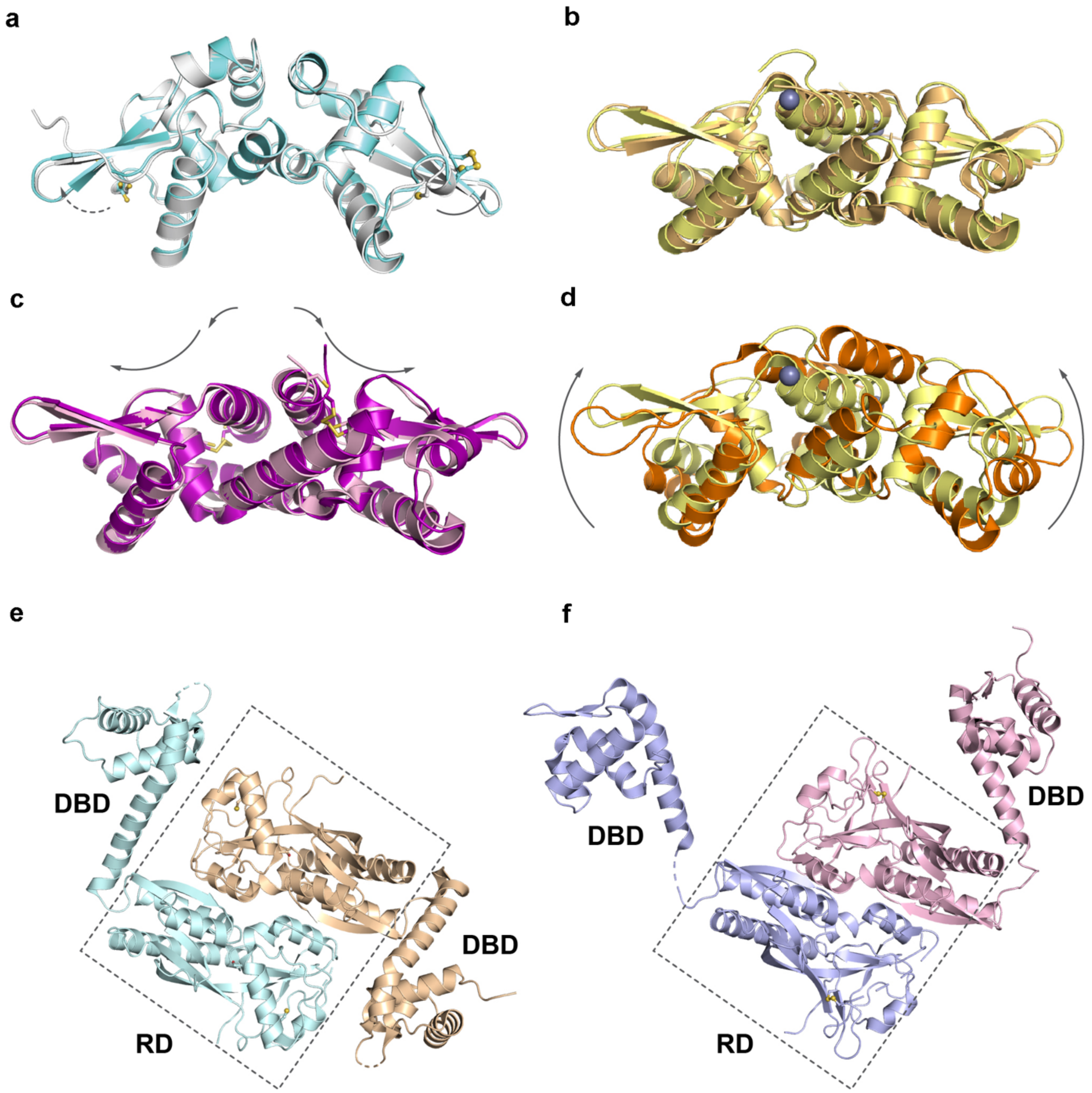

Supplementary Fig. 18. Comparison of RexT with other ArsR-SmtB members in different states suggests possible mechanism of regulation. (a) RexT is shown in its reduced (white) and oxidized states (cyan). Superposition of these two structures resulted an rmsd of 0.38 Å over 1258 atoms. The solid arrow highlights the local conformational change from the reduced state to the oxidized state in chain B that is observed in the crystal structures. The dashed arrow suggests a similar motion is possible in chain A if crystalline restraints were not in place. (b) *Sa*CzrA in its apo form[6] (wheat, PDB ID: 1R1U) and its Zn^{2+}-bound form[15] (yellow, PDB ID: 2M30, NMR structure). Superposition of two structures resulted an rmsd of 1.44 Å over 1287 atoms. (c) *Rc*SqrR[16] in its apo form (light pink, PDB ID: 6O8K) and tetrasulfide-bound form (dark pink, PDB ID: 6O8N). Superposition of the two structures resulted in an rmsd of 0.74 Å over 1185 atoms. Solid arrows suggest possible motions of the protein upon tetrasulfide formation. (d) *Sa*CzrA in its Zn^{2+}-bound form[15] (yellow) and DNA-bound form[17] (orange, PDB ID: 2KJB, NMR solution structure). Superposition of two structures resulted an rmsd of 4.39 Å over 2972 atoms. Arrows suggest the possible motion of *Sa*CzrA switching from the DNA-bound state to the Zn^{2+}-bound state. (e-f) The DNA binding domain (DBD) of *Cg*OxyR[18] shows a significant conformational change when OxyR transitions from the reduced state (PDB: 6G1D) to its oxidized state (PDB: 6G1B). The regulatory domains (RD) show little global movement. *Cg*OxyR is a tetramer, but a dimeric representation is shown here to better visualize the change in the DBD.

Supplementary Table 1. The sequence of DNA duplex probe used for EMSA and fluorescence anisotropy (FA)

DNA probe sequence EMSA	GCTTGCTAACAATCGCACAATCTCCAATCGCGCTGGATCACCTAAGG CATACAGCACTCCTGGTAAAGAAATATCTTTTCGGTCTGGATGATACA GAAATCTCATAGTTGCATTATCTCTGAAAATAAATTATATTTTTATT**<u>ATT CG</u>**ATAATATCGAATAAA**<u>CGAAT</u>**TAAGGGGCAAGTAATATGTCATCCAT TACAAATGTTACAGAAGCCACATTCAAGCAAGAAGTTCTGGATAGCAA CGTTCCAGTTCTAGTGGACTTTTGGGCCCCTTGGTGTGGCCCTTGTC GGATGGTAGCGCCGGTTGTGGATGAAGTTGCTAGCGA
DNA probe sequence FA	TTATT**<u>ATTCG</u>**ATAATATCGAATAAA**<u>CGAAT</u>**TAAGG

Note: The sequence is shown from 5' to 3' only. The palindromic DNA-binding sites[19] are in bold and underlined.

Supplementary Table 2. Binding constants of RexT and its variants to DNA duplex fluorescence probe

Protein	K_d (μM)	A_{max} (Initial)	A_{max} (Fitted)
WT	1.08±0.07	1.000	1.048±0.017
C40S	0.95±0.05	1.001	1.019±0.013
C41S	1.35±0.11	1.004	1.019±0.021
R26A	5.18±1.22	0.967	0.980±0.065
K50A	5.29±1.86	0.938	0.900±0.088
WT + 2.5 eq. Cd^{2+}	1.20±0.10	0.999	1.017±0.022

Supplementary Table 3. Primers used for mutagenesis

Variants		Primer sequence
C40S	F	5'-GCAAAGGCGAGCAGAGCTGCGCGGAATTC-3'
	R	5'-GAATTCCGCGCAGCTCTGCTCGCCTTTGC-3'
C41S	F	5'-GGCGAGCAGTGCAGCGCGGAATTCG-3'
	R	5'-CGAATTCCGCGCTGCACTGCTCGCC-3'
C105S	F	5'-CCGCTGCTGACCAGCCAACAGAGCG-3'
	R	5'-CGCTCTGTTGGCTGGTCAGCAGCGG-3'
R26A	F	5'-GGGCGACCCGGCGGCTCTGGAGATTGTT-3'
	R	5'-AACAATCTCCAGAGCCGCCGGGTCGCCC-3'
K50A	F	5'-TCGATTTTGCGATCGCGGCGAGCACCATGAGCAACC-3'
	R	5'-GGTTGCTCATGGTGCTCGCCGCGATCGCAAAATCGA-3'
R61A	F	5'-GCAACCACTTCAAAATTCTGGCTGAGAGCGGTGTGGT-3'
	R	5'-ACCACACCGCTCTCAGCCAGAATTTTGAAGTGGTTGC-3'
T68A	F	5'-GCGGTGTGGTTCTGGCCCGTAAGGAAGGC-3'
	R	5'-GCCTTCCTTACGGGCCAGAACCACACCGC-3'
Q74A	F	5'-CCGTAAGGAAGGCACCGCACACATCAACCGTCTG-3'
	R	5'-CAGACGGTTGATGTGTGCGGTGCCTTCCTTACGG-3'
H75A	F	5'-GTAAGGAAGGCACCCAAGCCATCAACCGTCTGCGTC-3'
	R	5'-GACGCAGACGGTTGATGGCTTGGGTGCCTTCCTTAC-3'
N77A	F	5'-GGCACCCAACACATCGCCCGTCTGCGTCGTGA-3'
	R	5'-TCACGACGCAGACGGGCGATGTGTTGGGTGCC-3'

Supplementary Table 4. Key ArsR-SmtB members highlighted in the sequence similarity network

Name	UniProt ID	PDB ID	Amino acid sequence	Sensory site
**Metal-binding**				
SeSmtB	P30340	1SMT, 1R22, 1R1T, 1R23	MTKPVLQDGETVVCQGTHAAIASELQAIAPEVAQSLAEFFAVLADPNR LRLLSLLARSELCVGDLAQAIGVSESAVSHQLRSLRNLRLVSYRKQGR HVYYQLQDHHIVALYQNALDHLQECR	α5C
MtSmtB	P9WMI5	/	MVTSPSTPTAAHEDVGADEVGGHQHPADRFAECPTFPAPPPREILDA AGELLRALAAPVRIAIVLQLRESQRCVHELVDALHVPQPLVSQHLKILK AAGVVTGERSGREVLYRLADHHLAHIVLDAVAHAGEDAI	α5C
SaCzrA	O85142	1R1U, 1R1V, 2KJB, 2KJC, 2M30, 4GGG, 6CDA, 6CDB	MSEQYSEINTDTLERVTEIFKALGDYNRIRIMELLSVSEASVGHISHQLN LSQSNVSHQLKLLKSVHLVKAKRQGQSMIYSLDDIHVATMLKQAIHHAN HPKESGL	α5C
BsCzrA	O31844	/	MTEFRETEQSAADLDEETLFLVAQTFKALSDPTRIRILHLLSQGEHAVN GIAEKLNLLQSTVSHQLRFLKNLRLVKSRREGTSIYYSPEDEHVLDVLQ QMIHHTQHD	α5C
ObBxmR	Q76L30	/	MSPKSAVNGAISQPHQENDTPTCDRAHLVDCSRVGDIQTQVLNTAKA QRMAEFFSLLGDANRLRLVSVLAKQELCVCDLAATLGMSESAVSHQL RAMRAMRLVSYRKVGRQVFYSLLDRHVLELYRAVAEHLDEES	α3N+α5C
SyZiaR	Q55940	/	MSKSSLSKSQSCQNEEMPLCDQPLVHLEQVRQVQPEVMSLDQAQQM AEFFSALADPSRLRLMSALARQELCVCDLAAAMKVSESAVSHQLRILRS QRLVKYRRVGRNVYYSLADNHVMNLYREVADHLQESD	α3N+α5C
NoAztR	Q8ZS91	/	MNKHKKKQDLDLIQSSDTPTCDTHLVHLDNVRSSQAQILPTDKAQQMA EIFGVLADTNRIRLLSALASSELCVCDLAALTKMSESAVCHQLRLLKAMR LVSYRREGRNVYYSLADSHVINLYRSLVENNTYATGTG	α3N
SaCadC	P20047	1U2W, 3F72	MKKKDTCEIFCYDEEKVNRIQGDLQTVDISGVSQILKAIADENRAKITYAL CQDEELCVCDIANILGVTIANASHHLRTLYKQGVVNFRKEGKLALYSLGD EHIRQIMMIALAHKKEVKVNV	α3N
MtNmtR	O69711	2LKP	MGHGVEGRNRPSAPLDSQAAAQVASTLQALATPSRLMILTQLRNGPLP VTDLAEAIGMEQSAVSHQLRVLRNLGLVVGDRAGRSIVYSLYDTHVAQ LLDEAIYHSEHLHLGLSDRHPSAG	α5C
MtKmtR	O53838	/	MYADSGPDPLPDDQVCLVVEVFRMLADATRVQVLWSLADREMSVNEL AEQVGKPAPSVSQHLAKLRMARLVRTRRDGTTIFYRLENEHVRQLVID AVFNAEHAGPGIPRHHRAAGGLQSVAKASATKDVG	α5C
MtCmtR	P9WMI9	2JSC	MLTCEMRESALARLGRALADPTRCRILVALLDGVCYPGQLAAHLGLTR SNVSNHLSCLRGCGLVVATYEGRQVRYALADSHLARALGELVQVVLA VDTDQPCVAERAASGEAVEMTGS	α4C

CtAntR	A0A096F4H2	6UVU	MALEKRNELPACSLKPSLQDRDLITSAEAGEVVVLFKVLANDTRLRLLH ALARSGGLCVTDLAAAVGMKPQAVSNQLQRLADRRILRAARCGNNIH YRIVDPCVLRMLELGLCLIEEAEQQAGG	α5
**Arsenite-binding**				
EcArsR	P15905	/	MLQLTPLQLFKNLSDETRLGIVLLLREMGELCVCDLCMALDQSQPKISR HLAMLRESGILLDRKQGKWVHYRLSPHIPSWAAQIIEQAWLSQQDDVQ VIARKLASVNCSGSSKAVCI	α3
AfArsR	B7J952	6J05	MEPLQDPAQIVARLEALASPVRLEIFRLLVEQEPTGLVSGDIAEHLGQPH NGISFHLKNLQHAGLVTVQREGRYQRYRAAMPVVRALVAYLTENCCHG TRDCALSGETRSPSVQEGNQ	α5C
CgArsR	A0A5H1ZR36	6J0E	MTTLHTIQLANPTECCTLATGPLSSDESEHYADLFKVLGDPVRLRILSQL AAGGCGPVSVNELTDLMGLSQPTISHHLKKMTEAGFLDRVPEGRVVLH RVRPELFAELRTVLQIGSMELLE	α3N
SpArsR	E6XPL1	/	MNIADMNVADMNVENAAKVLKELGHPTRLALFRLLVKGGYTGVAVGQL QEALQIPGSTLSHHISALMSAGIISQRREGRVLYCVPDYELLQGLVHFLQ DQCCSGQ	α5
BsAseR	P96677	/	MTIDVAAMTRCLKTLSDQTRLIMMRLFLEQEYCVCQLVDMFEMSQPAIS QHLRKLKNAGFVNEDRRGQWRYYSINGSCPEFDTLQLILHQIDQEDELL NHIKQKKTQACCQ	α3
MtRv2642	P71941	/	MSNLHPLPEVASCVVAPLVREPLNPPAAAEMAARFKALADPVRLQLLSS VASRAGGEACVCDISAGVEVSQPTISHHLKVLRDAGLLTSRRRASWVYY AVVPEALTVLSNLLSVHADAAPALGAPA	α3(N)
**Redox (?)**				
XfBigR	Q9PFB1	3PQJ, 3PQK	MVNEMRDDTRPHMTREDMEKRANEVANLLKTLSHPVRLMLVCTLVEGE FSVGELEQQIGIGQPTLSQQLGVLRESGIVETRRNIKQIFYRLTEAKAAQL VNALYTIFCAQEKQA	α2+α5
RcSqrR	D5AT91	6O8K, 6O8L, 6O8M, 6O8N, 6O8O	MDTAQDPQDDFDPEMGSDTDERCAALDAEEMATRARAASNLLKALAHE GRLMIMCYLASGEKSVTELETRLSTRQAAVSQQLARLRLEGLVQSRREG KTIYYSLSDPRAARVVQTVYEQFCSGD	α2+α5
VcHlyU	A0A3Q0L222	3JTH, 5ZNX	MNLKDMEQNSAKAVVLLKAMANERRLQILCMLHNQELSVGELCAKLQLS QSALSQHLAWLRRDGLVTTRKEAQTVYYTLKSEEVKAMIKLLHSLYCEE	α2+α5
VvHlyU	P52695	4OOI, 4K2E	MPYLKGAPMNLQEMEKNSAKAVVLLKAMANERRLQILCMLLDNELSVGE LSSRLELSQSALSQHLAWLRRDGLVNTRKEAQTVFYTLSSTEVKAMIELL HRLYCQANQ	α2+α5
EcYgaV	P77295	3CUO	MTELAQLQASAEQAAALLKAMSHPKRLLILCMLSGSPGTSAGELTRITGL SASATSQHLARMRDEGLIDSQRDAQRILYSIKNEAVNAIIATLKNVYCP	α2+α5
CgCyeR	Q8NLA4	/	MARKLEHPSLAEMNLNAIMFALSDPIRRQILSQLSCGHNDQACVAFELPV SKSTSTHHFRVLREAGLITQRYEGTAILSALRSEDMEARFPGLLTSVMRA EVEERNAADLPV	?

NoRexT	Q8YVV6	This work	MRFLYHPDRKDISLPGVLYALGDPARLEIVRLLASKGEQCCAEFDFAIAKS TMSNHFKILRESGVVLTRKEGTQHINRLRREDLETLFPGLLDAVLRSAQPL LTCQQSAIVK	α3
**Other**				
RfNolR	Q83TD2	4OMY, 4OMZ, 4ON0	MEHAMQPLSPEKHEEAEIAAGFLSAMANPKRLLILDSLVKEEMAVGALA NKVGLSQSALSQHLSKLRAQNLVSTRRDAQTIYYSSSSDSVMKILGALS EIYGAATSVVIEKPFVRKSA	?
BaPagR	O31178	2ZKZ	MTVFVDHKIEYMSLEDDAELLKTMAHPMRLKIVNELYKHKALNVTQIIQIL KLPQSTVSQHLCKMRGKVLKRNRQGLEIYYSINNPKVEGIIKLLNPIQ	?
MtRv0081	P9WMI7	6JMI	MESEPLYKLKAEFFKTLAHPARIRILELLVERDRSVGELLSSDVGLESSN LSQQLGVLRRAGVVAARRDGNAMIYSIAAPDIAELLAVARKVLARVLSD RVAVLEDLRAGGSAT	?
MtRv2034	O53478	/	MSTYRSPDRAWQALADGTRRAIVERLAHGPLAVGELARDLPVSRPAV SQHLKVLKTARLVCDRPAGTRRVYQLDPTGLAALRTDLDRFWTRALT GYAQLIDSEGDDT	?
SrDepR2	A0A2D1UFR6	/	MPENENAPRGEKTPHPRHPGDDDRKLHTVDARTLRAIAHPLRIRLLNA LREFGPATASKLGERLGESSGATSYHLRQLAESGLVEDAPELGKGRE RWWRAVHEGSIFESADFLAHTDPEVRGAIGVVMHEVATTHAQELNTW LGTMSEWPQEWRQSSDMSDFKVRLTPELARELSAKLHAVVESYRDV VPEDTEGSAVVRTHLHTFPRPSE	?
PfHSR	Q8U030	2P4W	MGEELNRLLDVLGNETRRRILFLLTKRPYFVSELSRELGVGQKAVLEHL RILEEAGLIESRVEKIPRGRPRKYYMIKKGLRLEILLTPTLFGSEMYEAK GVRKSPEYEQAKELIKSQEPINVKMRELAEFLHELNERIREIIEEKRELE EARILIETYIENTMRRLAEENRQIIEEIFRDIEKILPPGYARSLKEKFLNINI	?
PH1932	O59595	1ULY	MAKKVKVITDPEVIKVMLEDTRRKILKLLRNKEMTISQLSEILGKTPQTIY HHIEKLKEAGLVEVKRTEMKGNLVEKYYGRTADVFYINLYLGDEELRYI ARSRLKTKIDIFKRLGYQFEENELLNIMDRMSQKEFDATVRISKYIEEKE DALKDFSNEDIIHAIEWLSTAELARDEEYLELLKRLGSILKR	?

Note: Regulatory residues are colored. Cyan: residues in the Asp/Glu and His-rich metal binding motifs; Green: residues in the Cys-rich metal-binding motifs; Pink: Cys residues that bind arsenite or methyl-arsenite; Purple: Cys residues that bind an RSS or an unknown species; Yellow: Cys residues that bind an ROS; Gray, residues that resembles above-mentioned conserved motifs but do not exhibit any function and are likely the remnant of evolution.

Supplementary Table 5. 105 sequences of RexT-like regulators that may control Trx expression

UniProt ID	Organism	Taxonomic lineage	Amino Acid Sequence
A0A1Y0RN61	Nostocales cyanobacterium HT-58-2	Bacteria>Cyanobacteria>Nostocales	MRFLHHPDRKHISLAAVLYALGDPVRLEIVRRLALEGEHCCADFDFAIAKSTMSNHFKILRESGVVLSRKEGTQHINMLRKEDLEALFPGLLDAVLRAAKPLSICSSSSQQTASQQF
A0A1Z4GKH3	Anabaenopsis circularis NIES-21	Bacteria>Cyanobacteria>Nostocales>Aphanizomenonaceae>Anabaenopsis	MRFLYHPDKKDISLSAVLYALGDPVRLEIVRLLATKGEQCCADFDFAIAKSTMSNHFKILRESGVVLTHKEGTQHINQLRREDLEMLFPGLLEAVLRSAQPLFLCQKSMVNSQ
A0A161VQF9	Nodularia spumigena CENA596	Bacteria>Cyanobacteria>Nostocales>Aphanizomenonaceae>Nodularia	MKFLYHPDRKDISLPEVLYALGDPVRLEIVRLLATEGEQCCAGFDFAIAKSTMSNHFKILRESGVVLTHKEGTQHINRLRQADLEAMFPGLLDAVLQSAQPLRLYQQSTINTR
A0A218Q2F8	Nodularia sp. NIES-3585	Bacteria>Cyanobacteria>Nostocales>Aphanizomenonaceae>Nodularia	MKFLYHPDRKDISLPGVLYALGDPVRLEIVRLLASEGEQCCAGFDFAIAKSTMSNHFKILRESGVVLTHKEGTQHINRLRQEDLEALFPGLLDAVLGSAQPFIFYQQSSVNSR
A0ZIB8	Nodularia spumigena CCY9414	Bacteria>Cyanobacteria>Nostocales>Aphanizomenonaceae>Nodularia	MKFLYHPDRKDISLPGVLYALGDPVRLEIVRLLATQGEQCCAGFDFAIAKSTMSNHFKILRESGVVLTHKEGTQHINRLRQADLEAMFPGLLDAVLQSAQPLRLYQQSTINTR
A0A1Z4JT76	Calothrix brevissima NIES-22	Bacteria>Cyanobacteria>Nostocales>Calotrichaceae>Calothrix	MRFLYHPDRKDITLVGVLYALGDPVRLEIVRLLATKGEQCCAGFDFAIAKSTMSNHFKILRESGVVLTHKEGTQHINQLRREDLEARFPGLLDAVLQSAQPLNICATAGKYGA
A0A1Z4LZV3	Calothrix parasitica NIES-267	Bacteria>Cyanobacteria>Nostocales>Calotrichaceae>Calothrix	MRFLYHPEQKNISLAGVLYALGDPVRLEIVRQLATKKEQCCGDFDFAIAKSTMSNHFKILRESGIVLTRKEGTQHINTLRIEDLEELFPGLLEAILNSAQPFIISGKEPVVSQ
A0A2A2TC89	Calothrix elsteri CCALA 953	Bacteria>Cyanobacteria>Nostocales>Calotrichaceae>Calothrix	MRFLYHPDRKDMTLPGVLYALGDPVRLEIVRLLATRGEQCCAGFDFAIAKSTMSNHFKILRESGVVLTRKEGTQHINSLRRNDLDTLFPGLLNAILLAAQPLEICAAAKEAIAIANN
A0A3S5K375	Calothrix desertica PCC 7102	Bacteria>Cyanobacteria>Nostocales>Calotrichaceae>Calothrix	MRFLYHPDQKDMTLAGVLYALGDPVRLEIVRQLAVTGEQCCAGFDFAIAKSTMSNHFKILRESGVVLTRKEGTQHINTLRRNDLEVLFPGLLDAVLRSALPLEFTTTDREASREPAIASSN
K9UZI4	Calothrix sp. PCC 6303	Bacteria>Cyanobacteria>Nostocales>Calotrichaceae>Calothrix	MRFLYHPDRKDMTLAGVLYAFGDPVRLEIVRQLATIGEQCCAGFDFAIAKSTMSNHFKILRESGVVLTRKEGTQHINTLRRDDLEILFPGLLDAVLRSAQPLPICTEEAPEVPVLVSN
A0A0T7BYV6	Calothrix sp. 336/3	Bacteria>Cyanobacteria>Nostocales>Calotrichaceae>Calothrix>unclassified Calothrix	MRFLYHPDRKHISLAGVLYALGDPVRLEIVRQLATRGEQCCAGFEFAIAKSTMSNHFKILRESGVVWTRKEGTQHINSLRREELEVLFPGLLDAVLRSAQPMVNQESLVNS

A0A1Q4RSC0	Calothrix sp. HK-06	Bacteria>Cyanobacteria>Nostocales>Calotrichaceae>Calothrix>unclassified Calothrix	MRFLYHPDQKDMTLAGVLYALGDPVRLEIVRQLAVAGEQCCAGFDFAIAKSTMSNHFKILRESGVVLTRKEGTQHINTLRRNDLEVLFPGLLDAVLRSALPLEITTAEREASREPAIASSN
A0A1Z4FPD3	Calothrix sp. NIES-2098	Bacteria>Cyanobacteria>Nostocales>Calotrichaceae>Calothrix>unclassified Calothrix	MRFLYHPDRKDISLAGVLYALGDPVRLEIVRLLATKGEQCCAGFDFAIAKSTMSNHFKILRESGVVLTHKEGTQHINQLRREDLEALFPGLLDAVLRSAQPLNICASADKQTASKV
A0A1Z4H3E5	Calothrix sp. NIES-2100	Bacteria>Cyanobacteria>Nostocales>Calotrichaceae>Calothrix>unclassified Calothrix	MRFLYHPDRKDISLAGVLYALGDPVRLEIVRLLATKGEQCCAGFDFAIAKSTMSNHFKILRESGVVLTHKEGTQHINQLRREDLEALFPGLLDAVLQSAQPLNICVSADKQTASKV
A0A1Z4NEH8	Calothrix sp. NIES-3974	Bacteria>Cyanobacteria>Nostocales>Calotrichaceae>Calothrix>unclassified Calothrix	MRFLYHPDRKDISLAGVLYALGDPVRLEIVRQLATRGEQCCAGFDFAIAKSTMSNHFKILRESGVVLTRKEGTQHINILRRDDLNLLFPGLLDAILASAQSSTGFPPEERQSQNGISVVGAVQ
A0A1Z4R8I5	Calothrix sp. NIES-4101	Bacteria>Cyanobacteria>Nostocales>Calotrichaceae>Calothrix>unclassified Calothrix	MQGMRFLYHPDRKDMALAGVLYALGDPVRLEIVRLLATKGEQCCAGFDFAIAKSTMSNHFKILRESGVVLTRKEGTQHINTLRRDDLETLFPGLLDAILRSAIPLEVCASSKEVVGVGVGAANN
A0A1Z4SLC9	Calothrix sp. NIES-4105	Bacteria>Cyanobacteria>Nostocales>Calotrichaceae>Calothrix>unclassified Calothrix	MRFLYHPDQKDMTLAGVLYALGDPVRLEIVRQLAVTGEQCCAGFDFAIAKSTMSNHFKILRESGVVLTRKEGTQHINTLRRNDLEVLFPGLLDAVLRSAHPMEITTATDREPAIASSN
A0A2H2X5M9	Calothrix sp. NIES-4071	Bacteria>Cyanobacteria>Nostocales>Calotrichaceae>Calothrix>unclassified Calothrix	MRFLYHPDQKDMTLAGVLYALGDPVRLEIVRQLAVTGEQCCAGFDFAIAKSTMSNHFKILRESGVVLTRKEGTQHINTLRRNDLEVLFPGLLDAVLRSAHPMEITTATDREPAIASSN
K9PML9	Calothrix sp. PCC 7507	Bacteria>Cyanobacteria>Nostocales>Calotrichaceae>Calothrix>unclassified Calothrix	MRFLYHPDRKDISLPGVLYALGDPVRLEIVRLLATKGEQCCADFDFAIAKSTMSNHFKILRESGVVLSRKEGTQHINKLRFEDLDMLFPGLLDAVLQSAKPLGVCPSVVIGNRQIGK
A0A3S1AN91	Chlorogloeopsis fritschii PCC 6912	Bacteria>Cyanobacteria>Nostocales>Chlorogloeopsidaceae>Chlorogloeopsis	MRFLYHPERKNIYLTGVLYALGDPVRLEIVRQLAAKGEQCCADFDFAIAKSTMSNHFKILRESGVVWTRKEGTQHINSLRREDLEVLFPGLLDVVLRSAQPLMSQESTVISH
A0A1Z4UDG5	Aulosira laxa NIES-50	Bacteria>Cyanobacteria>Nostocales>Fortieaceae>Aulosira	MRFLYHPDRKDISLAGVLYALGDPVRLEIVRLLATQGKQCCAGFDFAIAKSTMSNHFKILRESGVVLTHKEGTQHINSLRREDLEALFPGLLDAVLRSAQPLNICTSANKQTASKVS
A0A328I829	Hapalosiphonaceae cyanobacterium JJU2	Bacteria>Cyanobacteria>Nostocales>Hapalosiphonaceae	MRFLYHPEQQHISLAGVLYALGDPVRLEIVRQLAVKGEQCCADFDFAIAKSTMSNHFKILRESGVIWTRKEGTQHINSLRREGLEELFPGLLDVVLRSPQPLITQESMVKSH
A0A0S3TLG6	Fischerella sp. NIES-3754	Bacteria>Cyanobacteria>Nostocales>Hapalosiphonaceae>Fischerella	MRFLFHPEQKHISLAGVLYALGDPVRLEIVRQLATKGEQCCADFDFAIAKSTMSNHFKILRESGVVWTRKEGTQHINSLRREDLEQLFPGLLDVVLRSAQPLLSQESMLKSR
A0A1U7H1B7	Fischerella major NIES-592	Bacteria>Cyanobacteria>Nostocales>Hapalosiphonaceae>Fischerella	MRFLFHPEQKHISLAGVLYALGDPVRLEIVRQLATKGEQCCADFDFAIAKSTMSNHFKILRESGVVWTRKEGTQHINSLRREDLEQLFPGLLDVVLRSAQPLLSQESMLKSR

A0A1Z4TTY8	Fischerella sp. NIES-4106	Bacteria>Cyanobacteria>Nostocales>Hapalosiphonaceae>Fischerella	MRFLYHPEQQHISLAGVLYALGDPVRLEVVRQLALKGEQCCADFDFAIAKSTMSNHFKILRESGVVWTRKEGTQHINSLRREDLEESFPGLLDVVLRAAQPLMIQESMVKNN
A0A2N6K1N1	Fischerella muscicola CCMEE 5323	Bacteria>Cyanobacteria>Nostocales>Hapalosiphonaceae>Fischerella	MRFLFHPEQKHISLAGVLYALGDPVRLEIVRQLATKGEQCCADFDFAIAKSTMSNHFKILRESGVVWTRKEGTQHINSLRREDLEQLFPGLLDVVLRSAQPLLSQESMLKSR
A0A2N6K9Y8	Fischerella thermalis CCMEE 5268	Bacteria>Cyanobacteria>Nostocales>Hapalosiphonaceae>Fischerella	MRFLFHPEQKHISLAGVLYALGDPVRLEIVRQLATKGEQCCADFDFAIAKSTMSNHFKILRESGVVWTRKEGTQHINSLRREDLEQLFPGLLDAVLRSAQPLLSQESMLKSR
A0A2N6KVH3	Fischerella thermalis CCMEE 5273	Bacteria>Cyanobacteria>Nostocales>Hapalosiphonaceae>Fischerella	MRFLFHPEQKHISLAGVLYALGDPVRLEIVRQLATKGEQCCADFDFAIAKSTMSNHFKILRESGVVWTRKEGTQHINSLRREDLEQLFPGLLDVVLRSAQPLLSQESMLKSR
A0A2N6LCH9	Fischerella thermalis CCMEE 5318	Bacteria>Cyanobacteria>Nostocales>Hapalosiphonaceae>Fischerella	MRFLFHPEQKHISLAGVLYALGDPVRLEIVRQLATKGELCCADFDFAIAKSTMSNHFKILRESGVIWTRKEGTQHINSLRREDLEQLFPGLLDAVLRSAQPLLSQESMLKSR
A0A2N6LYB7	Fischerella thermalis CCMEE 5205	Bacteria>Cyanobacteria>Nostocales>Hapalosiphonaceae>Fischerella	MRFLFHPEQKHISLAGVLYALGDPVRLEIVRQLATKGELCCADFDFAIAKSTMSNHFKILRESGVIWTRKEGTQHINSLRREDLEQLFPGLLDAVLRSAQPLLSQESMLKSR
A0A2N6MBZ3	Fischerella thermalis CCMEE 5201	Bacteria>Cyanobacteria>Nostocales>Hapalosiphonaceae>Fischerella	MRFLFHPEQKHISLAGVLYALGDPVRLEIVRQLATKGEQCCADFDFAIAKSTMSNHFKILRESGVVWTRKEGTQHINSLRREDLEQLFPGLLDAVLRSAQPLLSQESMLKSR
A0A2N6MDS7	Fischerella thermalis CCMEE 5330	Bacteria>Cyanobacteria>Nostocales>Hapalosiphonaceae>Fischerella	MRFLFHPEQKHISLAGVLYALGDPVRLEIVRQLATKGEQCCADFDFAIAKSTMSNHFKILRESGVVWTRKEGTQHINSLRREDLEQLFPGLLDVVLRSAQPLLSQESMLKSR
A0A0M0SU60	Hapalosiphon sp. MRB220	Bacteria>Cyanobacteria>Nostocales>Hapalosiphonaceae>Hapalosiphon>unclassified Hapalosiphon	MRLLYHPEQKHISLAGVLYALGDPVRLEVVRQLAVKGEQCCADFDFAIAKSTMSNHFKILRESGVIWTRKEGTQHINSLRREDLEKLFPGLLDVVLRSAQPLMSQESMVTTIQN
A0A4D9CHS4	Mastigocladus laminosus UU774	Bacteria>Cyanobacteria>Nostocales>Hapalosiphonaceae>Mastigocoladus	MRFLYHPEQQHISLAGVLYALGDPVRLEIVRQLAVKGEQCCADFDFAIAKSTMSNHFKILRESGVIWTRKEGTQHINSLRREDLEELFPGLLDVVLRSPQPLITQESMVKSH
A0A0V7ZSD4	Mastigocoleus testarum BC008	Bacteria>Cyanobacteria>Nostocales>Hapalosiphonaceae>Mastigocoleus	MRFLYHPDQRDITLPGVLYALGDPVRLEIVRLLAEKGEQCCADFDFVISREGSAPRTIAKSTMSNHFKILRESGVVFTRKEGTQHINKLRRQDLEGLFPGLLEAVLSSAKPLATSQGELTKQAASSFG
A0A4V2JNZ2	Westiellopsis prolifica IICB1	Bacteria>Cyanobacteria>Nostocales>Hapalosiphonaceae>Westiellopsis	MRFLYHPEQQHISLAGVLYALGDPVRLEIVRQLAVKGEQCCADFDFAIAKSTMSNHFKILRESGVIWTRKEGTQHINSLRREDLEELFPGLLDVVLRSPQPLITQESMVKSH
A0A1W5CB68	Anabaena sp. 39858	Bacteria>Cyanobacteria>Nostocales>Nostocaceae>Anabaena>Unclassified Anabaena	MRFLYHPDRKDISLPGVLYALGDPARLEIVRLLASKGEQCCAEFDFAIAKSTMSNHFKILRESGVVLTRKEGTQHINRLRYEDLEALFPGLLDAVLRSAQPLLTCQQSVIVK

A0A5Q0GAJ5	Anabaena sp. YBS01	Bacteria>Cyanobacteria>Nostocales >Nostocaceae>Anabaena>Unclassified Anabaena	MRFLYHPDRKDISLPGVLYALGDPARLEIVRLLASKGEQCCAEFD FAIAKSTMSNHFKILRESGVVLTRKEGTQHINRLRYEDLEALFPGL LDAVLRSAQPLLTCQQSVIVK
K9WYV3	Cylindrospermum stagnale PCC 7417	Bacteria>Cyanobacteria>Nostocales >Nostocaceae>Cylindrospermum	MRFLYHPDRKDICLPGVLYALGDPVRLEIVRLLATKGEQCCAEFN FAIAKSTMSNHFKILRESGVVLTHKEGTQHINQLRCEDLEALFPGL LGAVLRSAQPLLVESSTVKKSVSQMW
A0A1Z4QTA1	Cylindrospermum sp. NIES-4074	Bacteria>Cyanobacteria>Nostocales >Nostocaceae>Cylindrospermum>unclassified Cylindrospermum	MRFLYHPDRKDISLPGVLYALGDPVRLEIVRLLATEGEQCCAGFD FAIAKSTMSNHFKILRESGVVLTRKEGTHHINQLRSGDLEALFPGL LDAVLQSTQPLCVSPSTAKQGVSQMR
A0A0M4SSD5	Nostoc piscinale CENA21	Bacteria>Cyanobacteria>Nostocales >Nostocaceae>Nostoc	MRFLYHPDRKDISLPAVLYALGDPVRLEIVRLLASKGEQCCADFD FAIAKSTMSNHFKILRESGVVLTHKEGTQHINQLRREDLELLFPGL LEAVLRSAQPLLFQKSMAKSQ
A0A1C0VHB2	Nostoc sp. MBR 210	Bacteria>Cyanobacteria>Nostocales >Nostocaceae>Nostoc	MRFLYHPDRKDISLPAVLYALGDPVRLEIVRLLASKGEQCCADFD FAIAKSTMSNHFKILRESGVVLTHKEGTQHINQLRREDLELLFPGL LEAVLRSAQPLLCQKSMVKSQ
A0A1E2WIY2	Nostoc sp. KVJ20	Bacteria>Cyanobacteria>Nostocales >Nostocaceae>Nostoc	MRFLYHPDRKNISLPGVLYALGDPVRLEIVRLLATEGEQCCARFD FAIAKSTMSNHFKILRESGMVFTRKEGTQHINILRREDLEMLFPGL LDAVLKAAQPLPVVPASAKQTASRI
A0A1U7I345	Nostoc calcicola FACHB-389	Bacteria>Cyanobacteria>Nostocales >Nostocaceae>Nostoc	MRFLYHPDRKNISLPGVLYALGDPVRLEIVRLLASEGEQCCARFD FAIAKSTMSNHFKILRESGVVFTRKEGTQHINILRREDLEMLFPGL LDAVLKAAQPLSVDPASTKQTASTRGRGQGW
A0A1Z4HQ07	Nostoc carneum NIES-2107	Bacteria>Cyanobacteria>Nostocales >Nostocaceae>Nostoc	MRFLYHPDRKDISLAGVLYALGDPVRLEIVRLLATQGKQCCAGFD FAIAKSTMSNHFKILRESGVVLTHKEGTQHINSLRREDLEALFPGL LDAVLRSAQPLNICTSANKQTASKVS
A0A1Z4ICG4	Nostoc sp. NIES-2111	Bacteria>Cyanobacteria>Nostocales >Nostocaceae>Nostoc	MRFLYHPDRKDILLPGVLYALGDPVRLEIVRLLATKGEQCCADFD FAIAKSTMSNHFKILRESGVVMTRKEGTQHINRLRCEDLEVLFPG LLEAVLRSAQPLLICQQSIVVK
A0A1Z4L2J2	Nostoc linckia NIES-25	Bacteria>Cyanobacteria>Nostocales >Nostocaceae>Nostoc	MRFLYHPDRKNISLPGVLYALGDPVRLEIVRLLASEGEQCCAKFD FAIAKSTMSNHFKILRESGVVFTRKEGTQHINILRREDLEMLFPGL LDAVLKAARPLCVDPATTRQTASTKVGG
A0A1Z4S5G4	Nostoc sp. NIES-4103	Bacteria>Cyanobacteria>Nostocales >Nostocaceae>Nostoc	MRFLYHPDRKNISLPGVLYALGDPVRLEIVRLLATKGEQCCAEFD FAIAKSTMSNHFKILRESGVVLTHKEGTQHINQLRREDLEALFPGL LDAVLRSAQPLMICNQSKVPVKVVQRQSDR
A0A235HTK1	Nostoc sp. 'Peltigera membranacea cyanobiont' 213	Bacteria>Cyanobacteria>Nostocales >Nostocaceae>Nostoc	MRFLYHPDRKNISLPGVLYALGDPVRLEIVRLLATEGEQCCAQFD FAIAKSTMSNHFKILRESGIVLTRKEGTHHINILRREDLEMLFPGLL DAVLKAAQPLSICPGTKQTASRI
A0A235HY70	Nostoc sp. 'Peltigera membranacea	Bacteria>Cyanobacteria>Nostocales >Nostocaceae>Nostoc	MRFLYHPDRKNISLPGVLYALGDPVRLEIVRLLATEGEQCCAKFD FAIAKSTMSNHFKILRESGIVLTRKEGTHHINILRREDLEVLFPGLL DVVLKAAQPLPVEPASTKQTASRI

	cyanobiont' 210A		
A0A235IS72	Nostoc sp. 'Peltigera membranacea cyanobiont' 232	Bacteria>Cyanobacteria>Nostocales >Nostocaceae>Nostoc	MRFLYHPDRKNISLPGVLYALGDPVRLEIVRLLATEGEQCCAQFD FAIAKSTMSNHFKILRESGIVFTRKEGTHHINILRREDLEMLFPGLL DAVLKAAQPLPVSPASDKQTVSRI
A0A252DG98	Nostoc sp. T09	Bacteria>Cyanobacteria>Nostocales >Nostocaceae>Nostoc	MRFLYHPDRKDISLAGVLYALGDPVRLEIVRLLATQGEQCCAGFD FAIAKSTMSNHFKILRESGVVLTHKEGTQHINRLRREDLEALFPGL LDAVLQSAQPLNICASADKQTASTA
A0A252DU84	Nostoc sp. RF31YmG	Bacteria>Cyanobacteria>Nostocales >Nostocaceae>Nostoc	MRFLYHPDRKDISLAGVLYALGDPVRLEIVRLLATQGEQCCAGFD FAIAKSTMSNHFKILREAGVVLTHKEGTQHINRLRREDLEVLFPGL LDAVLQSAQPLNICASAAQQTASQV
A0A252E5W4	Nostoc sp. 106C	Bacteria>Cyanobacteria>Nostocales >Nostocaceae>Nostoc	MRFLYHPDRKDISLAGVLYALGDPVRLEIVRLLATQGEQCCAGFD FAIAKSTMSNHFKILREAGVVLTHKEGTQHINRLRREDLEVLFPGL LDAVLQSAQPLNICASADQQTASQV
A0A2C6W6J5	Nostoc linckia z16	Bacteria>Cyanobacteria>Nostocales >Nostocaceae>Nostoc	MRFLYHPDRKNISLPGVLYALGDPVRLEIVRLLASEGEQCCARFD FAIAKSTMSNHFKILRESGVVFTRKEGTQHINMLRREDLEMLFPG LLDAVLKAAQPLSVDSSSSKQTASSTSRRLGW
A0A2H6LDX9	Nostoc cycadae WK-1	Bacteria>Cyanobacteria>Nostocales >Nostocaceae>Nostoc	MRFLYHPDKKDISLSAVLYALGDPVRLEIVRLLATKGEQCCADFD FAIAKSTMSNHFKILRESGVVLTHKEGTQHINQLRREDLQMLFPG LLEAVLRSAQPLFLCQKSMVNSQ
A0A2I8AG27	Nostoc sp. CENA543	Bacteria>Cyanobacteria>Nostocales >Nostocaceae>Nostoc	MKFLYHPDRKDITLPGVLYALGDPVRLEMVCLLAAKGEQCCGDF DFAIAKSTMSNHFKILRESGVVFSRKEGTQHINRLRQEELEELFP GLLDAVLRSAKSIVNSQ
A0A2K8T0K6	Nostoc flagelliforme CCNUN1	Bacteria>Cyanobacteria>Nostocales >Nostocaceae>Nostoc	MRFLYHPDRKNISLPGVLYALGDPVRLEIVRRLATEGEQCCASFD FAIAKSTMSNHFKILRESGIVLTRKEGTQHINILRREDLEVLFPGLL DAILKAAQPLPVSPASIKQTASRN
A0A2L2NET1	Nostoc sp. 'Peltigera membranacea cyanobiont' N6	Bacteria>Cyanobacteria>Nostocales >Nostocaceae>Nostoc	MRFLYHPDRKNISLPGVLYALGDPVRLEIVRLLATEGEQCCAQFD FAIAKSTMSNHFKILRESGIVLTRKEGTHHINILRREDLEMLFPGLL DAVLKAAQPLSICPGTKQTASRI
A0A2L2NQS8	Nostoc sp. 'Lobaria pulmonaria (5183) cyanobiont'	Bacteria>Cyanobacteria>Nostocales >Nostocaceae>Nostoc	MRFLYHPDRKNISLPGVLYALGDPVRLEIVRLLATEGEQCCAKFD FAIAKSTMSNHFKILRESGIVFTRKEGTHHINILRREDLETLFPGLL DAVLKAAQPLPVSSVSDKQTASRI
A0A2R5FQ90	Nostoc commune NIES-4072	Bacteria>Cyanobacteria>Nostocales >Nostocaceae>Nostoc	MRFLYHPDRKNISLPGVLYALGDPVRLEIVRRLATEGEQCCASFD FAIAKSTMSNHFKILRESGIVFTRKSGTQHINILRREDLEVLFPGLL DAILKAAQPLPVSPASTKQTASRI

A0A2Z6CMH7	Nostoc sp. HK-01	Bacteria>Cyanobacteria>Nostocales >Nostocaceae>Nostoc	MRFLYHPDKKDISLSAVLYALGDPVRLEIVRLLATKGEQCCADFD FAIAKSTMSNHFKILRESGVVLTHKEGTQHINQLRREDLEMLFPGL LEAVLRSAQPLFLCQKSMVNSQ
A0A367QMX8	Nostoc sp. ATCC 43529	Bacteria>Cyanobacteria>Nostocales >Nostocaceae>Nostoc	MRFLYHPDRKNISLPGVLYALGDPVRLEIVRLLASEGEQCCARFD FAIAKSTMSNHFKILRESGVVFTRKEGTQHINILRREDLEMLFPGL LDAVLKAAQPLSVDPTSSKQTASTKV
A0A367RM59	Nostoc punctiforme NIES-2108	Bacteria>Cyanobacteria>Nostocales >Nostocaceae>Nostoc	MRFLYHPDRKNISLPGVLYALGDPVRLEIVRLLATEGEQCCAKFD FAIAKSTMSNHFKILRESGIVLTRKEGTHHINILRREDLEVLFPGLL DVVLKAAQPLPVSPVSTKQIASRI
A0A367RP87	Nostoc minutum NIES-26	Bacteria>Cyanobacteria>Nostocales >Nostocaceae>Nostoc	MRFLYHPDRKDISLPGVLYALGDPVRLEIVRLLATKGEQCCAEFD FAIAKSTMSNHFKILRESGVVLTHKEGTQHINQLRHEDLEALFPGL LDAVLRSAQPLLVCHQSTVNSR
A0A5P8VUG6	Nostoc sphaeroides CCNUC1	Bacteria>Cyanobacteria>Nostocales >Nostocaceae>Nostoc	MKFLYHPDRKNISLPGVLYALGDPVRLEIVRRLATEGEQCCASFD FAIAKSTMSNHFKILRESGIVLTRKEGTHHINMLRREDLEVLFPELL DAILKAAQPLPIEPASTKQTAFRI
A0A6P1KRA4	Nostoc sp. ATCC 53789	Bacteria>Cyanobacteria>Nostocales >Nostocaceae>Nostoc	MRFLYHPDRKNISLPGVLYALGDPVRLEIVRLLATEGEQCCAKFD FAIAKSTMSNHFKILRESGIVFTRKEGTHHINILRREDLEMLFPGLL DAVLKAAQPLPVSPASDKQTASRI
B2IWZ7	Nostoc punctiforme (strain ATCC 29133 / PCC 73102)	Bacteria>Cyanobacteria>Nostocales >Nostocaceae>Nostoc	MRFLYHPDRKNISLPGVLYALGDPVRLEIVRLLATEGEQCCAQFD FAIAKSTMSNHFKILRESGIVFTRKEGTHHINILRREDLEVLFPGLL DAVLKAAQPLPVSPASDKQTASRV
K9Q8R8	Nostoc sp. PCC 7107	Bacteria>Cyanobacteria>Nostocales >Nostocaceae>Nostoc	MRFLYHPDKKDISLSAVLYALGDPVRLEIVRLLATKGEQCCADFD FAIAKSTMSNHFKILRESGVVLTHKEGTQHINQLRREDLEMLFPGL LEAVLRSAQPLFLCQKSMVKSQ
K9QTD2	Nostoc sp. (strain ATCC 29411 / PCC 7524)	Bacteria>Cyanobacteria>Nostocales >Nostocaceae>Nostoc	MKFLYHPDRKNISLPGVLYALGDPVRLEIVRLLATKGEQCCADFD FAIAKSTMSNHFKILRESGVVLSRKEGTQHINKLRKEDLEALFPGL LDAVLRSAPPMLVCQ
Q8YVV6	Nostoc sp. (strain PCC 7120 / SAG 25.82 / UTEX 2576)	Bacteria>Cyanobacteria>Nostocales >Nostocaceae>Nostoc	MRFLYHPDRKDISLPGVLYALGDPARLEIVRLLASKGEQCCAEFD FAIAKSTMSNHFKILRESGVVLTRKEGTQHINRLRREDLETLFPGL LDAVLRSAQPLLTCQQSAIVK
A0A1Z4KEV0	Trichormus variabilis NIES-23	Bacteria>Cyanobacteria>Nostocales >Nostocaceae>Trichormus	MRFLYHPDRKDISLPGVLYALGDPARLEIVRLLASKGEQCCAEFD FAIAKSTMSNHFKILRESGVVLTRKEGTQHINRLRREDLETLFPGL LDAVLRSAQPLLTCQQSAIVK

Q3M3S7	Trichormus variabilis (strain ATCC 29413 / PCC 7937)	Bacteria>Cyanobacteria>Nostocales>Nostocaceae>Trichormus	MRFLYHPDRKDISLPGVLYALGDPARLEIVRLLASKGEQCCAEFDFAIAKSTMSNHFKILRESGVVLTRKEGTQHINRLRYEDLEALFPGLLDAVLRSAQPLLTCQQSVIVK
A0A1Z4M5J8	Fremyella diplosiphon NIES-3275	Bacteria>Cyanobacteria>Nostocales>Rivulariaceae>Microchaete	MRFLYHPDRKDISLAGVLYALGDPVRLEIVRLLATQGQQCCAGFDFAIAKSTMSNHFKILRESGVVLTHKEGTQHINRLRREDLEALFPGLLDAVLRSAQPLNICASANKQTASKVS
K9R5Y5	Rivularia sp. PCC 7116	Bacteria>Cyanobacteria>Nostocales>Rivulariaceae>Rivularia>unclassified Rivularia	MRFLYHPEQKNISLAGVLYALGDPVRLEIVRQLAKKKEQCCGDFDFAIAKSTMSNHFKILRESGVVLTRKEGTQHINTLRTEDLEELFPGLLEAVLNSAQPFVIAQEAVKQTV
A0A0C2Q9A4	Scytonema tolypothrichoides VB-61278	Bacteria>Cyanobacteria>Nostocales>Scytonemataceae>Scytonema	MRFLHHPDRKHISLAGVLYALGDPVRLEIVRRLAVKEEQCCADFDFAIAKSTMSNHFKILRESGVVLTRKEGTQHINMLRKEDLDALFPGLLDAVLRSAKPLSAKIRQENVIGSSTEPM
A0A139WRQ0	Scytonema hofmannii PCC 7110	Bacteria>Cyanobacteria>Nostocales>Scytonemataceae>Scytonema	MKFLYHPDKKNISLPGVLYALGDPVRLEIVRRLATEGEQCCGDFDFAIAKSTMSNHFKILRESGVILTRKEGTQHINILRREDLELLFPELLDVVLRSAKPLQLTMSRGLGIGDWG
A0A1Z4IW70	Scytonema sp. HK-05	Bacteria>Cyanobacteria>Nostocales>Scytonemataceae>Scytonema>unclassified Scytonema	MRLLYHPDRKHISLAGVLYALGDPVRLEIVRRLATQGEHCCADFDFAIAKSTMSNHFKILRESGVVLTRKEGTQHINMLRTEDLEALFPGLLDAVLRSAKPLCVGSSSYQKAGSQQVL
A0A1Z4Q644	Scytonema sp. NIES-4073	Bacteria>Cyanobacteria>Nostocales>Scytonemataceae>Scytonema>unclassified Scytonema	MRLLYHPDRKHISLAGVLYALGDPVRLEIVRRLATKGEHCCADFDFAIAKSTMSNHFKILRESGVVLTRKEGTQHINMLRTEDLDALFPGLLDAVLRSAKPLCVGSSSYQKAGSQ
A0A0C1N473	Tolypothrix bouteillei VB521301	Bacteria>Cyanobacteria>Nostocales>Tolypothrichaceae>Tolypothrix	MKFLYHPDKKNISLPGVLYALGDPVRLEIVRRLATEGEQCCGDFDFAIAKSTMSNHFKILRESGVILTRKEGTQHINILRREDLELLFPELLDVVLRSAKPLQLTVSRGLSVGDWG
A0A0C2QBQ3	Tolypothrix campylonemoides VB511288	Bacteria>Cyanobacteria>Nostocales>Tolypothrichaceae>Tolypothrix	MRLLYHPDRKHISLAAVLYALGDPVRLEIVRRLATQGEHCCADFDFAIAKSTMSNHFKILRESGVVLTRKEGTQHINMLRTEDLDALFPGLLDAVLRSAKPLCVGSSSYQKAGSQQVF
A0A1Z4MZN9	Tolypothrix tenuis PCC 7101	Bacteria>Cyanobacteria>Nostocales>Tolypothrichaceae>Tolypothrix	MRFLYHPDRKDISLAGVLYALGDPVRLEIVRLLATQGKQCCAGFDFAIAKSTMSNHFKILRESGVVLTHKEGTQHINSLRREDLEALFPGLLDAVLRSAQPLNICTSANKQTASKVS
A0A0D6KPE7	Tolypothrix sp. PCC 7601	Bacteria>Cyanobacteria>Nostocales>Tolypothrichaceae>Tolypothrix>unclassified Tolypothrix	MRFLYHPDRKDISLAGVLYALGDPVRLEIVRLLATQGQQCCAGFDFAIAKSTMSNHFKILRESGVVLTHKEGTQHINRLRREDLEALFPGLLDAVLRSAQPLNICASANKQTASKVS
A0A218QHS2	Tolypothrix sp. NIES-4075	Bacteria>Cyanobacteria>Nostocales>Tolypothrichaceae>Tolypothrix>unclassified Tolypothrix	MRFLYHPDRKDISLSGVLYALGDPVRLEIVRLLATVGAQPCAGFDFAIAKSTMSNHFKILRESGVVFTRKEGTQHINMLRRQDIDELFPGLLDAVLRSAQPMQICQEGTAVKSVSL
B0C3H5	Acaryochloris marina (strain MBIC 11017)	Bacteria>Cyanobacteria>Synechococcales>Acaryochloridaceae>Acaryochloris	MRLDIVNQLAASGELTCNAFDCEIAKSTLSHHFKILRESGVIYSRKEGTQHLNSLRREELDNLFPGLLASVLGSLSASA

A0A2W1K015	Acaryochloris sp. RCC1774	Bacteria>Cyanobacteria>Synechococcales>Acaryochloridaceae>Acaryochloris>unclassified Acaryochloris	MKLLYHPDCSQISLAGVLYALGDPVRLQVVQQLAADGELTCNAFDCDVAKSTMSHHFKILRESGVIRSRKEGTQHVNSLRQDELQELFPGLLDAVLQSSACGAVTG
A0A2W7A0F1	Leptolyngbya sp.	Bacteria>Cyanobacteria>Synechococcales>Leptolyngbyaceae>Leptolyngbya	MRPIPHPNCQDIALEGVLYALGDPVRLEIVQRLAANHELSCSDLDLGVAKSTLSHHFKILREAGVLHCRKQGTQHMNSLRRDDLEAAFPGLLDTILQAAQGIKGDR
A0A2W7BWP3	Leptolyngbya sp.	Bacteria>Cyanobacteria>Synechococcales>Leptolyngbyaceae>Leptolyngbya	MRPIHHPNCQDIALEGVLYALGDPVRLEIVQRLASNDELCCSDLDLGVAKSTLSHHFKILREAGVLHCRKQGTQHMNSLRRDDLEAAFPGLLDSILKAAQTQVER
A0A3M9Z491	Leptolyngbya sp. IPPAS B-1204	Bacteria>Cyanobacteria>Synechococcales>Leptolyngbyaceae>Leptolyngbya	MRLLYHPNQKDISLAGVLYALGDPVRLEIVRCLAEKGELPCAALDCDVPKSTMSHHLRVLREAGVLRCRKEGTQHINSLRQSDLDSLFPGLLEVVLRSMRPPALDGLPDAVATSLSIGANP
K9EQK9	Leptolyngbya sp. PCC 7375	Bacteria>Cyanobacteria>Synechococcales>Leptolyngbyaceae>Leptolyngbya	MRPISHPDTNQITLAEVLYALGDPVRLKIVKTIAQKGEQACRSCGGDEIAKSTLSHHFKILREAGIVHTEKVGTQHLNSLRVDELEAKFPGVLASVLAAADDGEVCEE
U9VKF5	Leptolyngbya sp. Heron Island J	Bacteria>Cyanobacteria>Synechococcales>Leptolyngbyaceae>Leptolyngbya	MRPIFHPDTTQITLADVLYALGDPVRLKIVKTIAQKGEQACRSCGGDDIAKSTLSHHFKILREAGIVHTQKVGTQHLNSLRLDELEARFPGVLPSVLAAATDIEVCDE
A0A2T1ES82	Stenomitos frigidus ULC18	Bacteria>Cyanobacteria>Synechococcales>Leptolyngbyaceae>Stenomitos	MRLLYHPDKKELSLAGVLYALGDPVRLEIVRRLAEKGELSCCEALEAQVAKSTLSHHFKVLRESGVLYCRKEGTQHMNSLRRADLDERFPGLLNTVLQAIEPLKA
A0A2W4WN42	Shackletoniella antarctica	Bacteria>Cyanobacteria>Synechococcales>Oculatellaceae>Shackletoniella	MRSIPHPNCQDIALEGVLYALGDPVRLEIVQRLAANHELSCSDLDLGVAKSTLSHHFKILREAGVLHCRKQGTQHMNSLRRDDLEAAFPGLLDTILQAAQGIKGDR
B8HSU5	Cyanothece sp. (strain PCC 7425 / ATCC 29141)	Bacteria>Cyanobacteria>Oscillatoriophycideae>Oscillatoriales>Cyanothecaceae>Cyanothece>unclassified Cyanothece	MRLIHHPDRKDISLAGVLYALGDPVRLEIVKRLAEKGELPCAALDVPAPKSTLSHHFKILREAGVLFCRKEGTQHLNSLRKDDLEARFPGLLAVVLQATHQPH
K9WKU2	Microcoleus sp. PCC 7113	Bacteria>Cyanobacteria>Oscillatoriophycideae>Oscillatoriales>Microcoleaceae>Microcoleus>unclassified Microcoleus	MRLLYHPDPKDISLAGVLYALGDPVRLEIVRRLATKGEQPCAAFDLAIARSTMSHHFKVLRESGVLYCRKEGTQHLNSLRREDLDALFPGLLEAVLQAAPQSLAVDHTPTPET
A0A1U7JBT6	Phormidium tenue NIES-30	Bacteria>Cyanobacteria>Oscillatoriophycideae>Oscillatoriales>Oscillatoriaceae>Phormidium	MRPIHHPNCQDIALEGVLYALGDPVRLEIVQRLASNDELSCSDLDLGVAKSTLSHHFKILREAGVLHCRKQGTQHMNSLRRDDLEAAFPGLLDSILKAAQTQVER
A0A2P8VLR4	filamentous cyanobacterium CCT1	Bacteria>Cyanobacteria	MRPIYHPDCKDITIEGVLYALGDPIRLEIVKRLAQEDEIPCSALDLPVAKSTLSHHFKILREAGVITCRKQGTQHLNSLRLQELQAKFPDLLDTVLRASNGTSPYAPLDRPGNH

A0A2P8VVL2	filamentous cyanobacterium CCT1	Bacteria>Cyanobacteria	MRPIHHPNCQDIALEGVLYALGDPVRLEIVQRLASNDELSCSDLDLGVAKSTLSHHFKILREAGVLHCRKQGTQHMNSLRRDDLEAAFPGLLDSILKAARAKVES
A0A2P8WDA8	filamentous cyanobacterium CCP5	Bacteria>Cyanobacteria	MRPIYHPDCKDITIEGVLYALGDPIRLEIVKRLAQEDEIPCSALDLPVAKSTLSHHFKILREAGVITCRKQGTQHLNSLRLQELQAKFPDLLDTVLRASNGTSPYAPLDRPGNH
A0A2T2W547	filamentous cyanobacterium CCP3	Bacteria>Cyanobacteria	MRPIHHPNPQDIALAGVLYALGDPVRLEIVQRLAANDELSCSDLDLGVAKSTLSHHFKILREAGVLHCRKQGTQHMNSLRRDDLEAAYPGLLDTILRSAQVSEAKP
A0A3B8JDY8	Cyanobacteria bacterium UBA8553	Bacteria>Cyanobacteria	MRLLYHPDKKDISLASVLYALGDPVRLEIVKQLAQKGELSCAALAMPVAKSTLSHHFKVLRESGVLHCRKEGTQHINSLRRQDLDDRFPGLLDTVLQSAMTTDN
A0A3D1P7C9	Cyanobacteria bacterium UBA11049	Bacteria>Cyanobacteria	MRLIYHPDRKDISLAGVLYALGDPVRLEIVKCLATKGEQPCAAMYFSIPKSTMSHHFKVLRESGTISCRKQGTQHLNSLRREDLDALFPGLLDAVLQAAGSYLQPSSGDGTD
A0A2S8SU85	Abditibacterium utsteinense	Bacteria>Abditibacteriota>Abditibacteria>Abditibacteriales>Abitibacteriaceae>Abditibacterium	MRELHHPSCESLSLPQVLHALSDPIRLCIAAQLDCRGDLPCGTFCESAAKSTMSHHFKVLRLAGVINQRTEGTSCFNTLRRADLDARFPGLLDAILRASNRW
A0A4Q3X1T0	Unclassified bacteria	Bacteria	MRELHHPSRDSISLPQVLHALSDPIRLCIVAELDNRGDLPCGTFCENAAKSTMSHHFKVLRLAGVILQRNEGTSCFNTIRRADLDARFPGLLDAIFQAESKNS
A0A4V1ZK08	Unclassified bacteria	Bacteria	MRELHHPPRECLELTQVLHALSDPIRLCIVRQLALGEALACGTFCETAPKSTMSHHFKVLRLSGVIHQRSEGTSCFNTLRRGDLDARFPGLLDAIVREEVGACGNSVWEEDSR

Supplementary References

1 Krissinel, E. & Henrick, K. Inference of macromolecular assemblies from crystalline state. *J Mol Biol* **372**, 774-797, doi:10.1016/j.jmb.2007.05.022 (2007).

2 Holm, L. & Rosenstrom, P. Dali server: conservation mapping in 3D. *Nucleic Acids Res* **38**, W545-549, doi:10.1093/nar/gkq366 (2010).

3 Mukherjee, D., Datta, A. B. & Chakrabarti, P. Crystal structure of HlyU, the hemolysin gene transcription activator, from Vibrio cholerae N16961 and functional implications. *Biochim Biophys Acta* **1844**, 2346-2354, doi:10.1016/j.bbapap.2014.09.020 (2014).

4 Ye, J., Kandegedara, A., Martin, P. & Rosen, B. P. Crystal structure of the Staphylococcus aureus pI258 CadC Cd(II)/Pb(II)/Zn(II)-responsive repressor. *J Bacteriol* **187**, 4214-4221, doi:10.1128/JB.187.12.4214-4221.2005 (2005).

5 Prabaharan, C., Kandavelu, P., Packianathan, C., Rosen, B. P. & Thiyagarajan, S. Structures of two ArsR As(III)-responsive transcriptional repressors: Implications for the mechanism of derepression. *J Struct Biol* **207**, 209-217, doi:10.1016/j.jsb.2019.05.009 (2019).

6 Eicken, C. *et al.* A metal-ligand-mediated intersubunit allosteric switch in related SmtB/ArsR zinc sensor proteins. *J Mol Biol* **333**, 683-695, doi:10.1016/j.jmb.2003.09.007 (2003).

7 Emsley, P. & Cowtan, K. Coot: model-building tools for molecular graphics. *Acta Crystallogr D Biol Crystallogr* **60**, 2126-2132, doi:10.1107/S0907444904019158 (2004).

8 Lee, S. G., Krishnan, H. B. & Jez, J. M. Structural basis for regulation of rhizobial nodulation and symbiosis gene expression by the regulatory protein NolR. *Proc Natl Acad Sci U S A* **111**, 6509-6514, doi:10.1073/pnas.1402243111 (2014).

9 Gerlt, J. A. Genomic Enzymology: Web Tools for Leveraging Protein Family Sequence-Function Space and Genome Context to Discover Novel Functions. *Biochemistry* **56**, 4293-4308, doi:10.1021/acs.biochem.7b00614 (2017).

10 Zallot, R., Oberg, N. O. & Gerlt, J. A. 'Democratized' genomic enzymology web tools for functional assignment. *Curr Opin Chem Biol* **47**, 77-85, doi:10.1016/j.cbpa.2018.09.009 (2018).

11 Crooks, G. E., Hon, G., Chandonia, J. M. & Brenner, S. E. WebLogo: a sequence logo generator. *Genome Res* **14**, 1188-1190, doi:10.1101/gr.849004 (2004).

12 Kumar, S., Stecher, G., Li, M., Knyaz, C. & Tamura, K. MEGA X: Molecular Evolutionary Genetics Analysis across Computing Platforms. *Mol Biol Evol* **35**, 1547-1549, doi:10.1093/molbev/msy096 (2018).

13 Stecher, G., Tamura, K. & Kumar, S. Molecular Evolutionary Genetics Analysis (MEGA) for macOS. *Mol Biol Evol* **37**, 1237-1239, doi:10.1093/molbev/msz312 (2020).

14 Jones, D. T., Taylor, W. R. & Thornton, J. M. The rapid generation of mutation data matrices from protein sequences. *Comput Appl Biosci* **8**, 275-282, doi:10.1093/bioinformatics/8.3.275 (1992).

15 Chakravorty, D. K. *et al.* Solution NMR refinement of a metal ion bound protein using metal ion inclusive restrained molecular dynamics methods. *J Biomol NMR* **56**, 125-137, doi:10.1007/s10858-013-9729-7 (2013).
16 Capdevila, D. A. *et al.* Structural basis for persulfide-sensing specificity in a transcriptional regulator. *Nat Chem Biol* **17**, 65-70, doi:10.1038/s41589-020-00671-9 (2021).
17 Arunkumar, A. I., Campanello, G. C. & Giedroc, D. P. Solution structure of a paradigm ArsR family zinc sensor in the DNA-bound state. *Proc Natl Acad Sci U S A* **106**, 18177-18182, doi:10.1073/pnas.0905558106 (2009).
18 Pedre, B. *et al.* Structural snapshots of OxyR reveal the peroxidatic mechanism of H2O2 sensing. *Proc Natl Acad Sci U S A* **115**, E11623-E11632, doi:10.1073/pnas.1807954115 (2018).
19 Ehira, S. & Ohmori, M. The redox-sensing transcriptional regulator RexT controls expression of thioredoxin A2 in the cyanobacterium Anabaena sp. strain PCC 7120. *J Biol Chem* **287**, 40433-40440, doi:10.1074/jbc.M112.384206 (2012).
